# Shelter selection in females of two scorpion species depends on shelter size and scent

**DOI:** 10.1007/s00359-024-01721-6

**Published:** 2024-11-02

**Authors:** Janina Hladik, Yorick Bailer, Harald Wolf, Torben Stemme

**Affiliations:** Institute of Neurobiology, Ulm University, Albert-Einstein-Allee 11, 89081 Ulm, Germany

**Keywords:** Arachnida, Chelicerata, Home choice, Sociality, Two choice decision, Chemosensation

## Abstract

Shelter selection is an important task in an animal’s life. Concerning scorpions, little is known on the evaluation of potential shelters and the importance of chemosensation. To address these issues, we conducted a two-choice shelter test in rectangular open field arenas to identify properties rendering shelters attractive for female scorpions of the species *E. italicus* and *M. eupeus*. Shelters varied in size (large, small) and scent (none, conspecific: male or female, attractive: prey, aversive: rosemary oil). Contact with the shelters was video-recorded under red light for 13 h, including the whole night phase. Results revealed a preference for larger shelters, with conspecific scent having minor or no influence. Striking differences occurred with regard to prey and rosemary oil scents. Prey scent was more attractive to *M. eupeus*, while rosemary oil did not act as a repellent. *E. italicus* was not very attracted by prey scent, but was repelled by rosemary oil. These findings might reflect the different habitats, semi-arid vs. Mediterranean climates: prey and rosemary are scarce in the semi-arid climate (habitat of *M. eupeus*), whereas they are abundant in the Mediterranean climate (habitat of *E. italicus*). We carried out impairment experiments to identify the main sensory organs responsible for the above observations. These are the pectines and pedipalps which function as mechano- and chemosensors. Scorpions could not detect size nor scent properly when either their pectines or pedipalps were impaired.

**Supplementary Information:**

The online version contains supplementary material available at 10.1007/s00359-024-01721-6.

## Introduction

Shelter selection is an important task for survival in vagile animals. In the resting phase, shelters protect from biotic and abiotic factors like predation and extreme temperatures (Polis and Farley 1980; Carlson and Rowe 2009). However, in scorpions, which spend most of their time in shelters, it remains largely unknown which qualities are preferred and which sensory inputs are needed to assess a shelter’s quality.

There are two types of shelters for scorpions (Review: Williams 1987): naturally existing ones - like gaps under stones, cracks in walls, burrows of other animals (e.g. Koch 1977; Kaltsas and Mylonas 2010) and burrows of different degrees of complexity dug by the scorpions themselves (e.g. Williams 1966; Review: Polis 1986; Polis et al. 1986). Stahnke (1966) grouped scorpions into digging “ground” scorpions (e.g. Vejovidae) and non-digging “bark” scorpions (e.g. Buthidae). In 1893, Pocock already wrote about the different behaviors of *Parabuthus capensis*, which he observed digging “very much as a rabbit”, and *Euscorpius carpathicus*, which never dug but “were usually to be found during the daytime under pieces of wood” (Pocock 1893).

In digging scorpions, e.g. the desert scorpion *Smeringurus mesaensis* (formerly *Paruroctonus mesaensis*), the females are stationary as they live in their burrows for longer time spans (Polis et al. 1985), which makes them a perfect study object for navigational questions (Gaffin et al. 2022). Additionally, females of *S. mesaensis* build larger burrows than males (Polis et al. 1986) and show a smaller home range (Polis et al. 1985). Digging-species, which are already well studied, build their home on their own even in the laboratory if given the right circumstances (Gaffin et al. 2022).

We focus here on females of non-digging scorpions, since there is very little knowledge on shelter selection in these species. Benton (1992a) investigated a colony of *E. flavicaudis* living in cracks of a wall at the docks of Sheerness, Kent and found that especially the females remained true to their shelters. Colombo (2006) confirmed the rare appearance of females of *E. italicus* outside of their shelters in gaps between or under rocks. As being the more stationary sex, females are suitable for shelter choice experiments because their need for an adequate shelter is supposedly more essential than in males. Female scorpions need extra protection by a suitable shelter, such as during rearing of offspring when they carry the litter on their back until the first molting (Shorthouse and Marples 1982; own observations).

### Influences on shelter selection

Non-burrowing species need an existing space to hide. Research on *Aegaeobuthus gibbosus* (formerly *Mesobuthus gibbosus*) and two *Vaejovis* species indicated that these scorpions prefer hideouts with larger diameters over smaller ones (Kaltsas et al. 2009; Becker and Brown 2016), and it can be assumed that the choice of shelter size may vary directly with the size of the animal. Besides abiotic factors like shelter size, temperature and humidity, biotic factors might play a role in shelter selection. Along these lines, scents of conspecifics or prey may attract or repel individual scorpions: Male *Smeringurus mesaensis* responded with precourtship behavior to substrate previously in contact with females (Gaffin and Brownell 1992). Similarly, male *Hadrurus arizonensis* followed a trail of a conspecific receptive female and avoided conspecific male scent (Melville et al. 2003). Male *Paruroctonus boreus* can not only detect conspecifics but discern animals of the same or a different population, possibly via pheromones (Miller and Formanowicz 2011). In a study by Steinmetz et al. (2004), male *Centruroides vittatus* reacted to chemical signals from females on substrate but not to airborne chemicals.

Beyond conspecific chemical cues, *Androctonus australis* and *Buthus occitanus* reacted to traces of prey chemicals on the ground through contact with their pectines, pedipalps or tarsi (Krapf 1986). *Leiurus quinquestriatus* in turn, did not prefer a compartment with prey odor over an empty one and avoided clove oil (Abushama 1964); Kelley et al. (2019) showed that *Centruroides vittatus* was deterred by rosemary oil, spending more time on sand treated with mineral oil instead.

### Scorpion sensory organs

For all these sensory tasks, scorpions use input from a variety of sensory organs (Abushama 1964; Root 1990; Brownell 2001; Review: Prévost and Stemme 2020). The most obvious scorpion sensory organs are the pectines, as well as numerous sensory hairs, and the lateral and median eyes. Although scorpion eyes show an extreme sensitivity to light, the resolution of their eyes has been suggested to be rather low (Locket 2001); as such, vision seems to be of only marginal importance in shelter evaluation.

Modalities like mechano- as well as chemosensation seem to play a more significant role than vision during the active night phase (Brownell 1977; Root 1990; Review: Prévost and Stemme 2020). Scorpions possess the unique comb-shaped, ventral pectines, which possess bimodal mechano- and chemosensory sensilla (Cloudsley-Thompson 1955; Foelix and Müller-Vorholt 1983; Brownell 1989; Gaffin and Brownell 1997; Wolf 2008, 2017; Knowlton and Gaffin 2011). Further important sensory structures are chemo- and mechanosensory sensilla, especially on those structures that are in direct contact with the substrate, namely the pedipalps and the tarsi of walking legs. However, information regarding modality, sensitivity, and function of these sensilla is rather scarce. Nevertheless, some studies have addressed sensilla associated with the denticle subrows (Lowe and Fet 2024) and the constellation arrays on the pedipalps (Fet et al. 2006), as well as sensory hairs and hair-like sensilla on scorpion tarsi as well as the tarsal organ (Foelix and Schabronath 1983). Furthermore, behavioral studies provided first evidence that the pedipalps perceive not only mechanosensory but also contact or airborne chemosensory cues (Abushama 1964; Nisani et al. 2018). This suggests that pedipalps are likely to play an important role in shelter evaluation according to their exposed position on the body surface.

Scorpions prefer narrow shelters where their dorsal mechanoreceptors have contact with the shelter ceiling, especially when hiding after an escape response (Torres and Heatwole 1967). Tactile input is also involved in positive thigmotaxis and negative thigmokinesis when scorpions stay in contact with objects (Abushama 1964), which may help guide the animals to the opening of a retreat. Tactile information via mechanosensory input may also play a role in determination of shelter size.

To evaluate the quality of a shelter, the animals need to collect and assess input by sensory structures. In this context, chemo- and mechano-sensation on the pedipalps or pectines appear to be the most important. As such, we performed a set of impairment experiments in which sensory input from either pecten or pedipalp sensilla or both was abolished.

### Aims of the study

Since most knowledge on scorpion shelter choice is anecdotal, we have taken an experimental approach to provide a more complete picture. We will focus on two important properties of a shelter, namely size and scent. Along these lines, we check the responses to differently attractive and aversive scents, and we try to identify the most significant chemosensory structures involved in shelter selection. By studying two species from different habitats, we address the potential for generalization in non-burrowing species and dependencies on environmental conditions.

### Hypotheses

1. According to literature data (Kaltsas et al. 2009; Becker and Brown 2016) showing that certain scorpions prefer hideouts with larger diameters over smaller ones, we expect tested scorpions to opt for the larger shelter when confronted with a choice between large and small (but otherwise neutral) shelters.
2. We hypothesize that female scorpions of *E. italicus* and *M. eupeus* are interested in conspecific male scent and therefore spend more time in contact with male-scented shelters compared to neutral shelters, no matter the size.
3. Female test scorpions will be less interested in female-scented shelters compared to male-scented shelters, when directly compared.
4. The scent of a conspecific female animal might indicate a suitable shelter, when tested separately against neutral shelters. Nevertheless, a larger neutral shelter might be more favorable than a small female scented shelter, as there is possibly not enough space for two competitive animals.
5. Concerning prey scent, these shelters should be more interesting than a neutral shelter, as food is a basal need.
6. While food scent should be attractive, rosemary scent should be aversive to scorpions, as demonstrated by Kelley et al. (2019). Therefore, a neutral shelter should be more attractive than a rosemary-scented shelter. This should hold true even for small neutral shelters tested against large rosemary-scented shelters, although scorpions usually prefer larger shelters.
7. We expect longer total contact times during the night with the shelter that the scorpion will chose as a final hiding place: More interest leads to longer investigation time and to the final decision in favor of the shelter.
8. Finally, with impaired sensory organs, scorpions will not be able to detect scent and size as well as in the intact state. Hence, we expect the animals to spend more time in contact with the shelters to discriminate scents and sizes. We further expect the ratio of chosen shelters to tend toward 50:50.

## Materials and methods

### Animals and keeping

In this study, we focus on two species from different habitats. *Euscorpius italicus* (Herbst 1800) lives in Mediterranean climates of southern Europe (France, Switzerland, Italy, Greece), Southeastern Europe (Slovenia, Croatia, Montenegro, Albania) and around the Black Sea (Fet et al. 2004; Colombo 2006; Vignoli and Salomone 2008; Fet 2010). The species takes advantage of human settlements where it hides in natural stone or brick walls (Crucitti et al. 1998, 2005; Braunwalder 2005; Colombo 2006). *Mesobuthus eupeus* (Koch 1839) dwells in semi-arid areas with compacted sandy soil (Fet 1980). The species’ distribution ranges from northern Africa and around the Black Sea to western China (Farzanpay 1988; Kovařík 1999; Gromov 2001; Teruel 2002; Karataş and Karataş 2003; Shi et al. 2007; Kovařík et al. 2011, 2022).

Forty female *Euscorpius italicus* (+ 4 males and 1 female for perfuming shelters) were collected in Northern Italy (Bolzano area) and another 10 females (+ 2 females for perfuming shelters) were bought from a private breeder. Animals were kept individually in fauna boxes (18 × 11 × 12.5 cm, Exo Terra) filled with about 1 cm of gardening soil. The boxes were enriched with a terracotta shard as shelter and with a small dish of water. Water was replenished and the soil moistened three times a week. Scorpions were fed house crickets (*Acheta domestica*) every other week. Keeping and testing of the animals took place under natural photoperiod at the University of Ulm, Germany (approx. 48° northern latitude) and at ambient room temperatures (approx. 22–24 °C).

Twenty-seven female *Mesobuthus eupeus* (+ 3 males and 3 females for perfuming shelters) were obtained from a pet shop (thepetfactory.de) and kept individually in fauna boxes (18 × 11 × 12.5 cm, Exo Terra) filled with about 1 cm of sand substrate. All other keeping details were the same as for *E. italicus*. However, animals were kept under artificial reverse 14:10 light: dark regime (lights off at 10 am) and at about 26 °C room temperature.

The sex of all tested animals was confirmed either by having larvae during the time of the experiments, which confirmed female status, or by sacrificing them after completion of the experiments and checking their internal reproductive organs.

### Experimental design

Experiments with *E. italicus* were carried out between July and November. Forty females were tested in a suite of two-choice situations. In each test, two shelters were offered in a rodent polycarbonate cage (42.5 × 27.6 × 15.3 cm, 1290D Eurostandard Typ III, Tecniplast) filled with gardening soil about 2 cm deep (Universal Gartenerde, ASB Grünland Helmut Aurenz GmbH, Stuttgart-Weilimdorf, Germany). The soil was flattened and levelled with a spoon to prevent the animals from hiding under loose substrate. Terracotta plant saucers (Blumentopf-Fabrik Spang GmbH and Co KG, Ransbach-Baumbach, Germany) were used as shelters, 10.8 cm diameter serving as “large” and 6.3 cm diameter as “small” hideouts. For each test, the two shelters were placed on the soil of the test box in random positions, each about 5 cm away from the short side. A level tablespoonful of the respective test soil – neutral or scented (see below) – was put beneath the shelters. As *E. italicus* rarely digs, a small entrance hole was formed in the gardening soil substrate under the shelters’ rim, facing the middle of the box. These preparations were completed one hour before the beginning of the tests, which took place over night (13 h, 5 pm to 6 am, daylight saving time) and were recorded via a sports camera (SONY, HDR-AS50) under red light (2 bulbs: FLAIR LED Lampe E27/4 W A 60 Filament rot, Hornbach Baumarkt AG, Bornheim, Germany). Five individuals, one per test box, and thus five tests, were filmed in parallel on the same video. In the morning, at around 10:30 am, the final decision of the scorpions was noted, the soil was removed, and test boxes and test shelters were cleaned with tap water, demineralized water, and finally by 70% ethanol. At the beginning of the light phase, two additional lamps with full day light spectrum (2 bulbs: Natural Light, 25 W, Exo Terra) were switched on.

*Mesobuthus eupeus* were tested in the same experimental setup as described above for *E. italicus*. Only temperature (26 °C) and time regimen (artificial 14:10 day/night) cycle were adjusted to the species’ needs, and the test boxes were filled with playground sand 2 cm deep (Sahara Spielsand, WECO GmbH & Co. KG, Leer, Germany). This was done 20 h before testing, so the sand had time to dry, which avoided burying by the scorpions. About 1 h before the experiment, the sand surface was smoothed using a ruler that had been cleaned with 70% ethanol, and shelters were added. Experiments started about 30 min before the night cycle commenced and scorpion behavior was recorded for 13 h. Six individuals, one per test box, and therefore six tests were filmed in parallel on the same video. Final decisions of the scorpions were noted the next day, about 9 h after the end of the experiment. Like in the setup for *E. italicus*, the sand was removed from the boxes and the boxes and shelters were washed with tap and demineralized water, followed by cleaning with 70% ethanol.

### Test details

The tests consisted of 16 different pairings of 2 shelter size options and 6 scent options (compare Table 1). Scent options were: neutral (= no scent added), female conspecific, male conspecific, prey (= cricket, *Acheta domestica*), rosemary oil, mineral oil. Shelters were perfumed in the following manner. Four individuals of *E. italicus* (two males, two females) were kept overnight individually in larger fauna boxes (23 × 15.5 × 17 cm, Exo Terra) to perfume cleaned shelters in the box for the next test round. Scorpions were fed in separate feeding boxes to avoid the scent of prey on shelters and substrate. For the setup of *M. eupeus*, six *M. eupeus* individuals (three males, three females) were used in the same way to perfume test shelters with conspecific scent. The prey-scented test shelters were likewise perfumed by keeping about 15 crickets in a larger fauna box with soil or sand as substrate, depending on tested scorpion species, and adding the shelters for the tests to be carried out the following day. For the rosemary oil experiments, we followed the procedure by Kelley et al. 2019. Rosemary-scented test shelters were perfumed the day before the experiment. 300 mL of dried soil, or 200 mL of dried sand, respectively, were put into a plastic zip-lock bag. A mixture of 1 volume part mineral oil and 4 volume parts rosemary oil was made. 1 mL oil mix per 100 mL substrate was added into the zip-lock bag and mixed thoroughly for 30 s. A single clean test shelter per bag was added. It was made sure that the shelters were covered in substrate for about 22 h. The mineral oil-scented shelters were perfumed likewise. Scented substrate was used two days in a row to perfume shelters and tested against dried substrate of same age and initial pool.

**Table 1:** Combinations of shelter size and associated shelter scents used for testing *E. italicus* and *M. eupeus*. “Large” terracotta saucers had diameters of 10.8 cm, “small” ones 6.3 cm. Scents were added by conspecific male or female individuals, crickets as prey items, or indirect impregnation with scent oils (see Materials and methods). Female individuals encountered one shelter combination per test round, and they were tested only once per combination and in different chronological order to avoid habituation

	Shelter 1: size, scent	Shelter 2: size, scent
Test 1	large, neutral	small, neutral
Test 2	large, male	large, neutral
Test 3	large, male	small, neutral
Test 4	large, female	large, neutral
Test 5	large, female	small, neutral
Test 6	small, male	large, neutral
Test 7	small, female	large, neutral
Test 8	large, male	large, female
Test 9	small, male	small, neutral
Test 10	small, female	small, neutral
Test 11	small, male	small, female
Test 12	large, prey	large, neutral
Test 13	small, prey	large, neutral
Test 14	large, rosemary oil	large, neutral
Test 15	large, rosemary oil	small, neutral
Test 16	large, mineral oil	large, neutral

Every animal was tested once per test. Tests were named by the properties of the used shelters: first the size, followed by the additional scent, e.g. a large, neutral shelter “large, neutral” was tested against a small, prey-scented shelter “small, prey”. All tests were carried out in different chronological orders per animal to avoid effects of habituation. Animals were allowed to rest for at least 36 h between tests. We minimized the chance of a scorpion choosing shelter by other than the intended factors by changing the position of boxes (rotating system), as well as alternating tests per box, and by varying the boxes where individuals were placed.

In *M. eupeus*, we did not observe, nor suspect, a general seasonality of breeding and mating, at least under laboratory conditions. By pooling the data for each scorpion species separately over the whole testing period we tried to compensate for possible differences in behavior due to seasonality, which is indeed present in *Euscorpius* (Benton 1992a, b, 1993; Braunwalder 2005).

### Impairment experiments

Based on the results for the aforementioned experiments, two further experiments per species were performed with impaired sensory organs. Test animals were divided into two groups, and anesthetized with CO_2_ and cooled for about one hour in the fridge. One group had their pectines severed as close to the base as possible. The other group had the pedipalps covered in blue acrylic touch-up paint (MOTIP DUPLI, Dupli-Color Lackstift No. 120 − 0100). These manipulations were completed at least 5 days prior to the first test to ensure proper healing of wounds and acclimatization to the new situation. Details of pecten amputation and evidence of natural wound healing can be found in Stemme (2024) . Integrity of the pedipalp covers was visually checked before each test. Test procedures were the same as stated above. Twenty females (10 per group) of *E. italicus* were tested on small neutral vs. large neutral shelters and on small neutral vs. large rosemary oil-scented shelters. Eighteen individuals of *M. eupeus* (9 per group) were tested in the neutral setup (small neutral vs. large neutral) and on small prey-scented vs. large neutral shelters. In intact animals, these test combinations showed the most obvious differences, making further comparison of impairment effects easier. When all animals had participated in both tests, the sensory organ not yet manipulated was impaired, as described above. The previous two test rounds were repeated with both pectines and pedipalps impaired after at least 5 days for wound healing and acclimatization. As there was no difference between the tested animals anymore, all animals per species were considered one group, which resulted in a larger sample size.

### Data acquisition and statistics

To achieve better contrast of animals against substrate on the videos, scorpions were marked on the metasoma the day before the initial experiment. This was done by anesthetizing individuals with CO_2_ and cooling them for about half an hour in the fridge. *E. italicus* were painted with a white acrylic touch-up paint pen (MOTIP DUPLI, Dupli-Color Lackstift No. 0-0750). *M. eupeus* were painted with a blue acrylic touch-up paint pen (MOTIP DUPLI, Dupli-Color Lackstift No. 120 − 0100). Color was refreshed as needed, one day before the upcoming experiment. Videos were analyzed manually using the VLC media player 3.0.12 (https://www.videolan.org/vlc/), the images being enlarged as needed by interactive zoom. Time lapse was used for scanning the material and speed was slowed down to 0.67 times when animals were close to shelters.

The parameters “on top” and “underneath shelter” were analyzed. “On top” was defined by clearly visible contrast of any body part against the shelter, ending when there was no visible contrast anymore. The scorpion was considered “underneath shelter” as soon as approx. half of the body (marking) disappeared under the shelter, ending when the scorpion was visible in total. It is important that the scorpions leave the shelter completely because sometimes they tend to wait in their shelters for prey with anterior body parts exposed. It was not possible for the animals to fulfill both parameters at the same time, as there was only one opening to the shelter consisting of a hole beneath the saucers and also due to scorpion anatomy. We also verified that tested scorpions investigated each shelter at least once. Individuals that did not make contact with either shelter were removed from statistical analysis since it remained uncertain whether they had been aware of the two shelter options. Videos that could not be analyzed, for example, due to accidentally lost animal markings, were also removed from further analysis. These are the reasons for different numbers of animals per test, as we did not repeat tests for individuals to prevent habituation effects.

“Final hiding” was defined as the shelter where the scorpion was hidden in the morning, at the end of the experiment. In a pilot experiment with *E. italicus*, we had observed that *E. italicus* rarely switches hiding places during the day. Therefore, we interpreted the morning shelter as final decision of the tested scorpion.

**Fig. 1:**
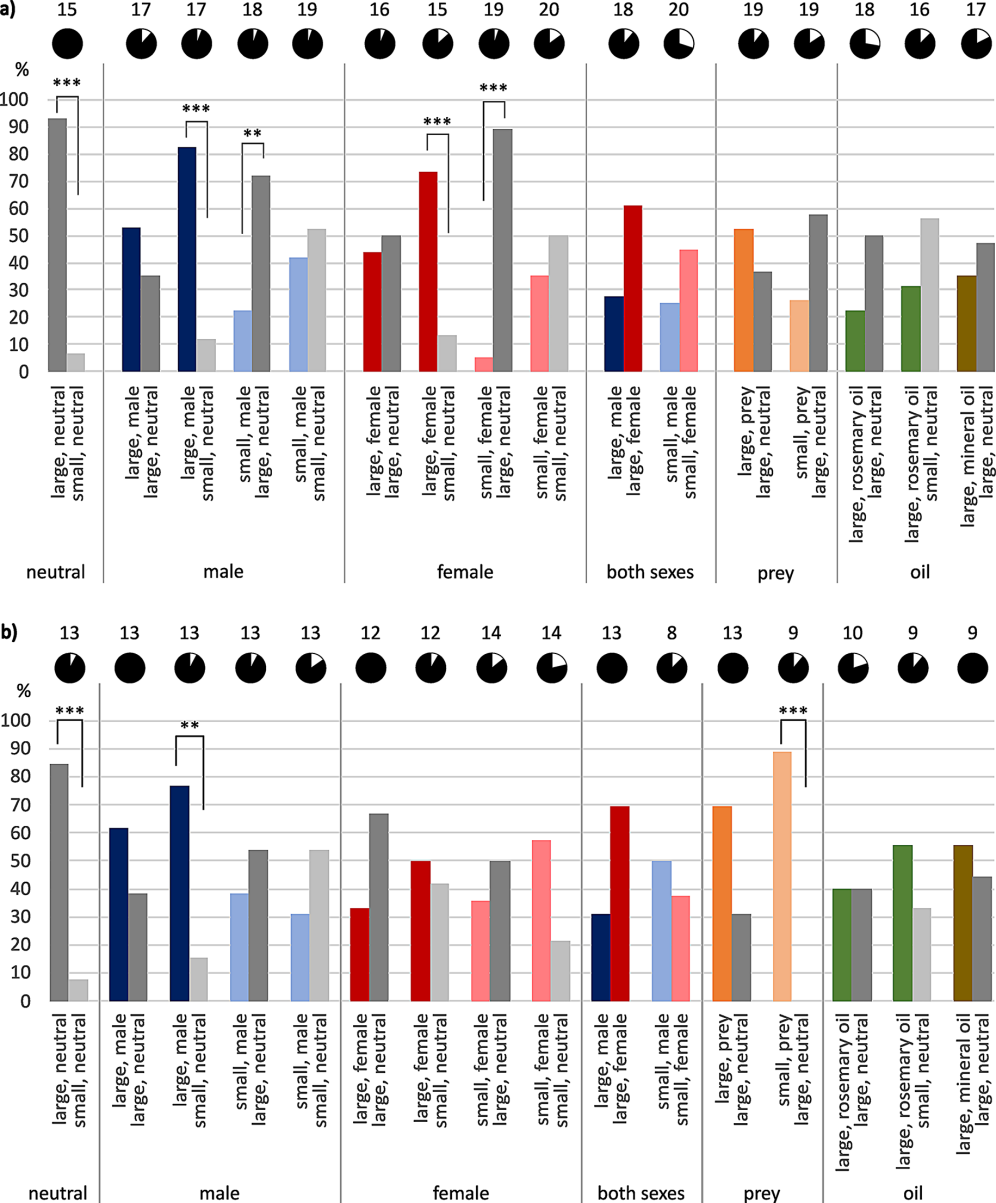
Final shelter choice in pairwise comparison. Females of (**a**) *E. italicus*, (**b**) *M. eupeus.* Ordinates show choices in percent. Pie charts above choice pairs indicate hidden (black) versus non-hidden (white) animal numbers, sample sizes noted above pie charts. Tests are sorted by stimulus condition (neutral = grey, male scent = blue, female scent = red, scent of both sexes, prey scent = orange, rosemary oil = green, mineral oil = brown) and shelter size (large-large = dark colors; large-small, small-large, small-small = light colors). Statistically significant differences indicated by asterisks: ** *P* < 0.01, *** *P* ≤ 0.001

**Fig. 2:**
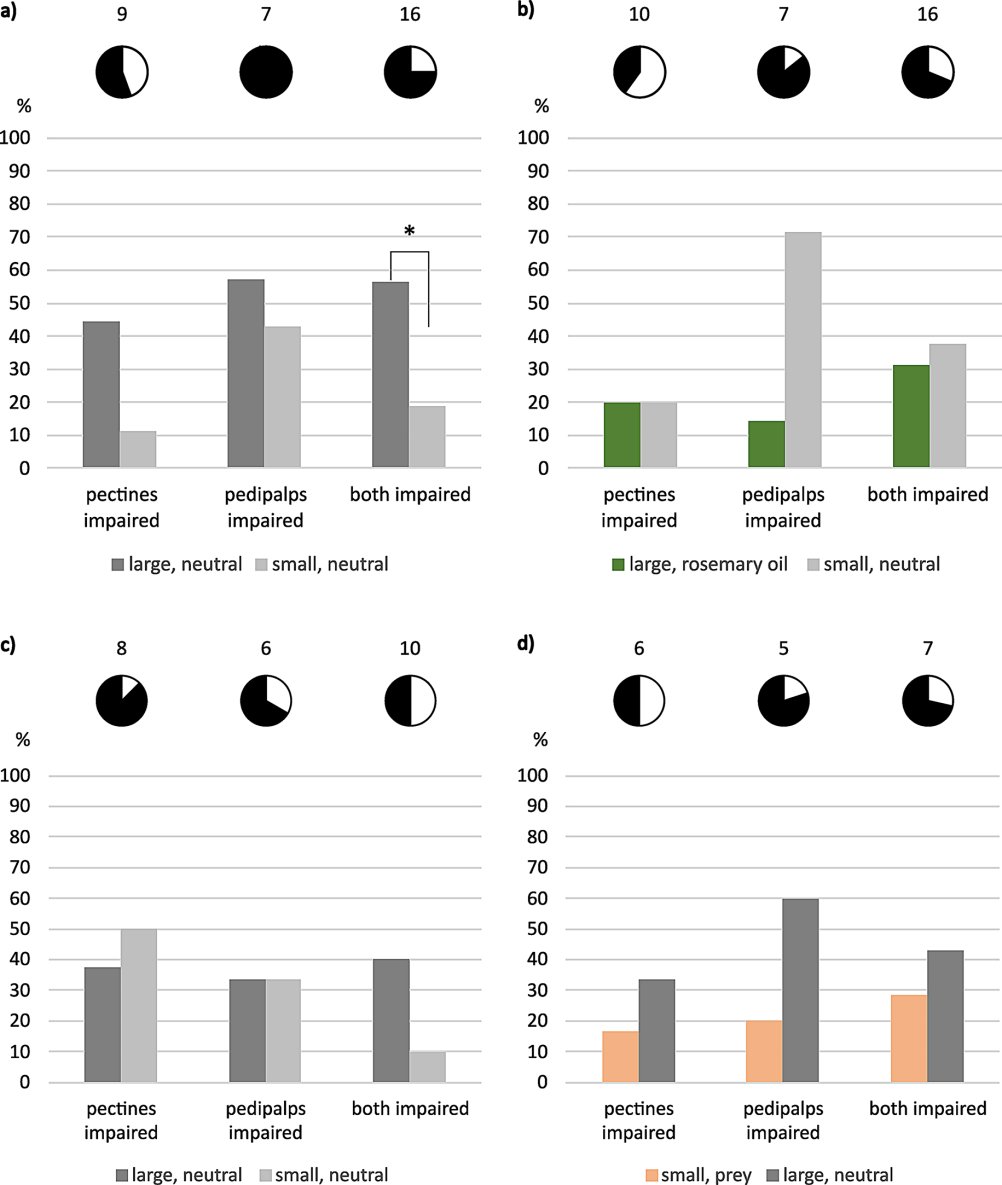
Final shelter choice of female (**a-b**) *E. italicus* and (**c-d**) *M. eupeus*. One or two sensory organs impaired, ordinates show choices in percent. (**a**, **c**) Large neutral tested against small neutral shelters, (**b**) large shelters perfumed with rosemary oil tested against small neutral shelters, (**d**) small prey-scented shelters tested against large neutral shelters. Pie charts show portions of hidden (black) versus non-hidden (white) animal numbers, sample sizes on top. Statistically significant difference indicated: * *P* < 0.05. Color code: grey = neutral, orange = prey, green = rosemary oil; dark colors = large shelters, light colors = small shelters

Statistics were performed with SigmaPlot 11.0. We counted the total numbers of hidden animals per final hiding place per test and compared them via Fisher-Exact Test. We also counted the numbers of animals not hidden and displayed the ratio of hidden versus not hidden individuals by pie charts (see Figs. 1 and 2). For shelter contact times, we calculated the times of “on top” and “underneath” the shelter, as well as the total contact times and the respective medians using Excel (Microsoft Office 2016). We checked the time data of each shelter pair for Normality (Kolmogorov-Smirnov-Test), followed by a Wilcoxon Signed Rank test in case there was no normal distribution. If the normality test passed, we performed a paired t-test. For better comparison, especially between species, we calculated percentages for all data sets, either with regard to total numbers of hiding place choices or of total observation time. We also calculated standard deviations and standard errors of medians with Excel for all categories of contact times (Online Resource: Tables S1, S3, S5, S7). All statistical results were further tabulated by species (Online Resource: Tables S2, S4, S6, S8).

Finally, we compared the time per shelter per animal with the final hiding place choice. If a scorpion spent longer total time at the shelter that was also its final hiding place, this was assigned the value “true”, otherwise “false”. We summed all “true” values of all tests and calculated the percentage true values of all final hiding place choices for each species. This yielded the true rate for the hypothesis “if a scorpion spends longer time in contact with a shelter, the scorpion will choose this shelter as its final hiding place”.

## Results

### Final hiding place

First, we describe results regarding the final hiding place, where the tested female scorpions were found in the morning, at the end of the experiment. We interpret this shelter to be the scorpion’s final decision where to hide during the day (see Data acquisition). Tested shelters are abbreviated by naming their properties, e.g. a small, prey-scented hide-out is referred to as “small, prey”.

### Intact females

When comparing neutral shelters, that is, without chemosensory cues present, female *E. italicus* significantly preferred larger hides over smaller ones (93.3%: 6.7%, Fisher-Exact: *P* < 0.001, *n* = 15, Fig. 1a). They also preferred the larger shelter when they had to choose between a large neutral shelter and a small one provided with conspecific scent. Tested animals chose the large neutral hideout significantly more often, independent of male (72.2%: 22.2%, Fisher-Exact: *P* = 0.005, *n* = 18, 1 animal not hidden) or female scent (89.5%: 5.3%, Fisher-Exact: *P* < 0.001, *n* = 19, 1 animal not hidden) being associated with the smaller shelter. This preference for larger shelters held true when the larger hiding place was scented and the small one remained neutral (male scent: 82.4%: 11.8%, Fisher-Exact: *P* < 0.001, *n* = 17, 1 animal not hidden; female scent: 73.3%: 13.3%, Fisher-Exact: *P* = 0.001, *n* = 15, 2 animals not hidden). In a direct comparison of male and female scent, females decided in favor of female scent, no matter if both retreats were large (61.1%: 27.7%, *n* = 18, 2 animals not hidden) or small (45.0%: 25.0%, *n* = 20, 6 animals not hidden). If scented and neutral shelters were both large ones, tested females hid more often underneath the male-scented than the neutral one (52.9%: 35.3%, *n* = 17, 2 animals not hidden), and preferences were about equal in the case of neutral versus female scent (50.0%: 43.8%, *n* = 16, 1 animal not hidden). When both shelters were small ones, females tended to hide in the neutral one compared to female-scented shelters (50.0%: 35.0%, *n* = 20, 3 animals not hidden) and they were nearly indifferent concerning male scent versus neutral shelters (42.1%: 52.6%, *n* = 19, 1 animal not hidden). When tested females had to decide between “large, neutral” and “large, prey”, they hid more often in the prey-scented one (36.8%: 52.6%, *n* = 19, 2 animals not hidden, Fig. 1a). In comparison with “small, prey”, the animals tended to prefer “large, neutral” (26.3%: 57.8%, *n* = 19, 3 animals not hidden). Rosemary scent on a large retreat appeared to have an aversive effect as the tested animals hid more often underneath the neutral retreat, no matter whether it was large (22.2%: 50.0%, *n* = 18, 5 animals not hidden) or small (31.3%: 56.3%, *n* = 16, 2 animals not hidden).

Female scorpions of *M. eupeus* hid significantly more often underneath a large final hiding place than underneath a small one in neutral scent comparison (84.5%: 7.7%, Fisher-Exact: *P* < 0.001, *n* = 13, 1 animal not hidden; Fig. 1b). Even when one of the shelters was fragrant with conspecific scent, they showed a tendency toward the larger shelter. Especially when the larger hideout was scented by males, females significantly favored that one over a small neutral one (76.9%: 15.4%, Fisher-Exact: *P* = 0.003, *n* = 13, 1 animal not hidden). If there were two large shelters, females hid more often in the male-associated hiding place than in the large neutral one (68.5%: 31.5%, *n* = 13), and more often underneath “large, neutral” than “large, female” (66.7%: 33.3%, *n* = 12). Interestingly, it was the other way round for small versus small retreat tests: Here, females apparently preferred female scent over neutral (57.1%: 21.4%, *n* = 14, 3 animals not hidden), and neutral over male scent (53.8%: 30.8%, *n* = 13, 2 animals not hidden). In direct comparison, female scent was chosen more often when presented against male scent on large shelters (69.2%: 30.8%, *n* = 13). Prey scent was preferred in both cases tested. On large final hiding places (69.2%: 30.8%, *n* = 13), and even on small shelters, where the preference was significant (88.9%: 0.0%, Fisher-Exact: *P* < 0.001, *n* = 9, 1 animal not hidden) when tested against large neutral shelters (Fig. 1b). Rosemary oil applied on large retreats (40%: 40%, *n* = 10, 2 animals not hidden) appeared to have almost no influence on shelter choice, as they were chosen as final hiding place almost equally compared to a large neutral shelter. When the neutral shelter was small, however, tested representatives tended toward the larger rosemary-scented shelter (33.3%: 55.6%, *n* = 9, 1 animal not hidden).

### Impaired females

With one impaired sensory organ, *E. italicus* still chose “large, neutral” over “small, neutral”, although results were no longer significant (Fig. 2a). In case of both organs impaired, tested animals significantly preferred the larger shelter compared to the smaller one (56.2%: 18.8%, Fisher-Exact: *P* = 0,039, *n* = 16, 4 animals not hidden). Female scorpions showed a clear tendency toward the small neutral retreat compared to “large, rosemary oil” when their pedipalps had been impaired (Fig. 2b).

With their pectines or their pedipalps severed, *M. eupeus* showed no significant preferences anymore: in neutral comparison, the scorpions seemed to make no difference between large and small shelters. Concerning prey odor, tested animals with impaired pectines preferred “large, neutral” over “small, prey” (Fig. 2d). This tendency was even more obvious with impaired pedipalps. When both sensory organs were impaired, tested individuals preferred the larger (unscented) shelter in both tests.

### Females not hidden

Intact animals appeared to select a final hiding place less often when both offered retreats were small (Fig. 1, Tables S2, S6). It further mattered whether both small hidings were perfumed with conspecific scent or just one of them was perfumed with female or male scent, tested against neutral. Added rosemary oil in a large-large test combination also increased the number of females not choosing a final shelter. There was a tendency towards more animals not hidden at all when the sensory organs had been impaired (Fig. 2, Tables S4, S8). Especially when the pectines had been cut, many animals refused to choose a final hiding place.

### Contact times

Recording duration of the experiments was 13 h, thus covering the entire night phase. The longest median total contact times with shelters during these hours ranged from over 6 h to nearly no contact at all. For better comparison, we calculated the contact times as percentages of total observation time, rounded to one decimal place. The situation “on top” was defined by clearly visible contrast of any body part against the shelter, ending when there was no visible contrast anymore. Scorpions were considered “underneath shelter” as soon as approximately half of the body disappeared underneath the shelter, ending when the scorpion was visible in total. “Total contact time” was calculated as the sum of “on top” and “underneath shelter”.

### *E. italicus*, median total contact times

**Fig. 3:**
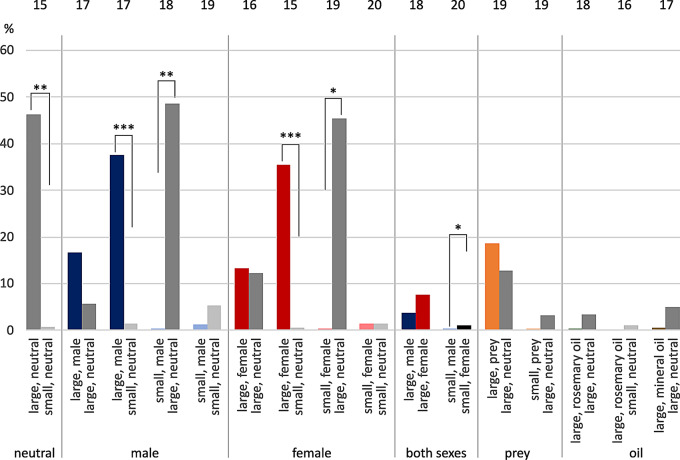
Median total times in contact with shelters in female *E. italicus*. Ordinate, percent of total observation time. Tests are sorted by stimulus (neutral, male, female, both sexes, prey, rosemary oil, mineral oil) and size of shelters (large-large, large-small, small-large, small-small). Statistically significant differences are indicated by asterisks: * *P* < 0.05, ** *P* < 0.01, *** *P* ≤ 0.001. Error bars omitted for clarity (see Tab. S1). Sample sizes on top. Color codes: grey = neutral, blue = male, red = female, orange = prey, green = rosemary oil, brown = mineral oil; dark colors = large shelters, light colors = small shelters

Regarding neutral size comparison, larger shelters reached significantly longer median total contact times than small ones (46.2%: 0.7%, paired t-test: t = -3.892, *n* = 15, *P* = 0.002, Fig. 3). When conspecific scent was added, tested females showed median 37.7% total contact time for the large male-scented shelter, which was significantly longer than 1.4% for the small neutral shelter (Wilcoxon Signed Rank Test: W = 127.000, *n* = 17, *P* = 0.001). They also showed a significantly longer median contact time with “large, female” compared to “small, neutral” (35.5%: 0.4%, paired t-test: t = -3.477, *n* = 15, *P* = 0.004). The larger hideout was also favored in the comparison of “large, neutral” versus small with conspecific scent, no matter whether associated with male (48.6%: 0.4%, paired t-test: t = 3.643, *n* = 18, *P* = 0.002) or female scent (45.4%: 0.3%, Wilcoxon Signed Rank Test: W = -122.000, *n* = 19, *P* = 0.012). For male versus female scent added to small shelters, there was a statistical significance concerning contact times in favor of female scent (1.1%: 0.3%, Wilcoxon Signed Rank Test: W = -120.000, *n* = 20, *P* = 0.024).

The large hideout with prey scent was probed for longer times than the large neutral shelter (Fig. 3). When the prey scent was associated with the small shelter and tested against “large, neutral”, median total contact times where about equal. For large shelters scented with rosemary oil, there were almost no median total contact times compared to neutral shelters.

### *E. italicus*, median times “on top” and “underneath”

Walking across the presented shelters will give the tested female scorpions a first impression of the options provided. Therefore, we wanted to know the time the tested animals spent “on top” of a hiding place in comparison to the time span exploring the shelters in more detail by hiding “underneath” the plant saucers serving as shelter. We also calculated contact time in percentages of total recording time.

**Fig. 4:**
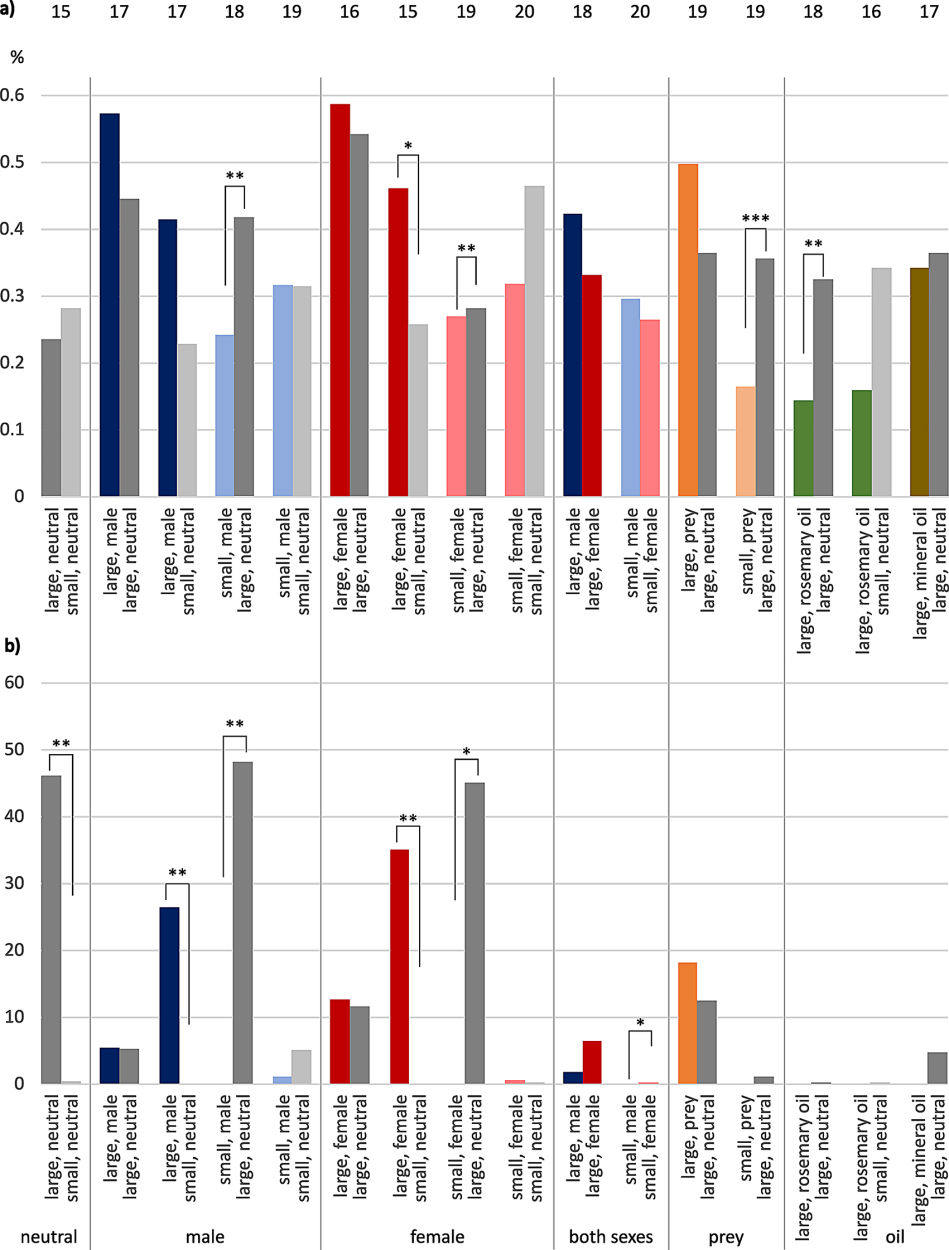
Median times in contact with shelters in female *E. italicus*, plotted separately for times (**a**) on top of shelter and (**b**) underneath shelter. Ordinate, percent of total observation time. Tests are sorted by stimulus (neutral, male, female, both sexes, prey, rosemary oil, mineral oil) and shelter size (large-large, large-small, small-large, small-small). Statistically significant differences indicated by asterisks: * *P* < 0.05, ** *P* < 0.01, *** *P* ≤ 0.001. Error bars omitted for clarity (see Table S1). Sample sizes on top. Color codes: grey = neutral, blue = male, red = female, orange = prey, green = rosemary oil, brown = mineral oil; dark colors = large shelters, light colors = small shelters

Median times on top of hiding places ranged below 1% of total recording time (Fig. 4a). As these time spans were comparably short and did not add much to the total contact times, we only describe striking differences like changed proportions. The first difference to the previously presented results was a longer contact time with the small neutral shelter compared to the larger neutral shelter (0.3%: 0.2%, *n* = 15). Significant differences in favor of large neutral hideouts were observed in four instances: in tests against “small, male” (0.4%: 0.2, Wilcoxon Signed Rank Test: W = -133.000, *n* = 18, *P* = 0.002), “small, female” (0.28%: 0.27%, Wilcoxon Signed Rank Test: W = -138.000, *n* = 19, *P* = 0.004), “small, prey” (0.4%: 0.2%, Wilcoxon Signed Rank Test: W = -152.000, *n* = 19, *P* = 0.001), and “large, rosemary oil” (0.3%: 0.1%, Wilcoxon Signed Rank Test: W = -129.000, *n* = 18, *P* = 0.003). Also, the median time on top of shelters was significantly longer for “large, female” (0.5%) compared to small neutral hideouts (0.3%, paired t-test: t = -4.475, *n* = 15, *P* < 0.001).

Median contact times underneath shelters were not much different concerning statistical significances compared to median total contact times, as noted above (Fig. 4b, Table S2).

### *E. italicus*, impaired sensory organs

**Fig. 5:**
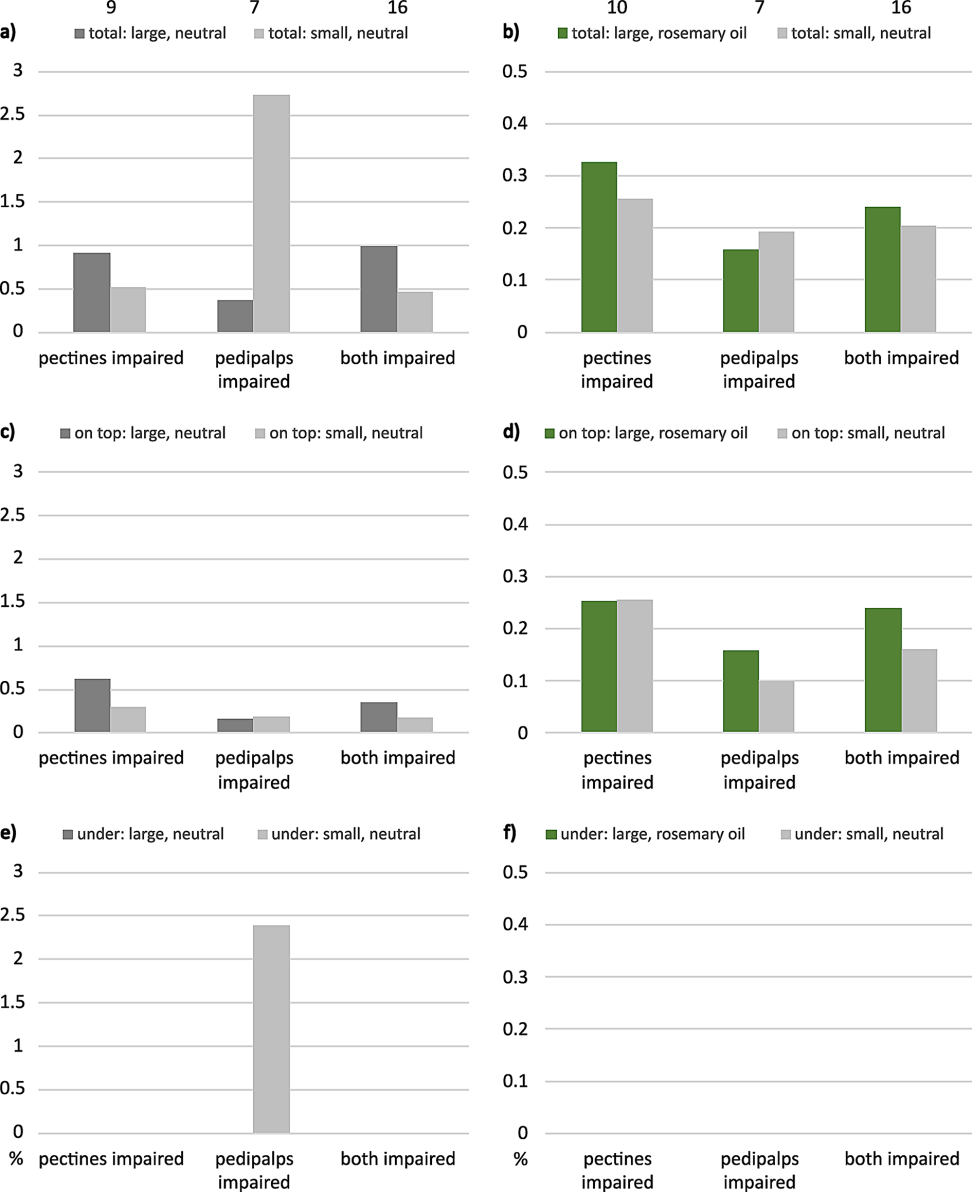
Median contact times of female *E. italicus* with impaired sensory organs. Ordinate, percent of total observation time. Shelter combinations, (**a**, **c**, **e**) large versus small neutral, and (**b**, **d**, **f**) “large, rosemary oil” versus “small, neutral”. (**a**, **b**) Total time in contact with shelter, (**c**, **d**) time on top of shelter, (**e**, **f**) time spent underneath shelter. Error bars omitted for clarity (see Table S3). Sample sizes on top. Color codes: grey = neutral, green = rosemary oil; dark colors = large shelters, light colors = small shelters

With impaired sensory organs, median contact times mostly ranged below 1% of the total observation time. The only exception was the neutral comparison of shelter size when the tested animals had their pedipalps impaired (Fig. 5a). In this case, maximum median contact times reached 2.7% of total observation time. Proportions of the median total contact times still showed the tendency in favor of “large” over “small” in both test situations, neutral and rosemary oil. Only when the pedipalps were impaired, tested females spent longer time in contact with the small neutral shelter, especially in the neutral test combination. In the latter case, the longer median time in contact with the small shelter seems to result from the time spend underneath the small shelter.

### *M. eupeus*, median total contact times

**Fig. 6:**
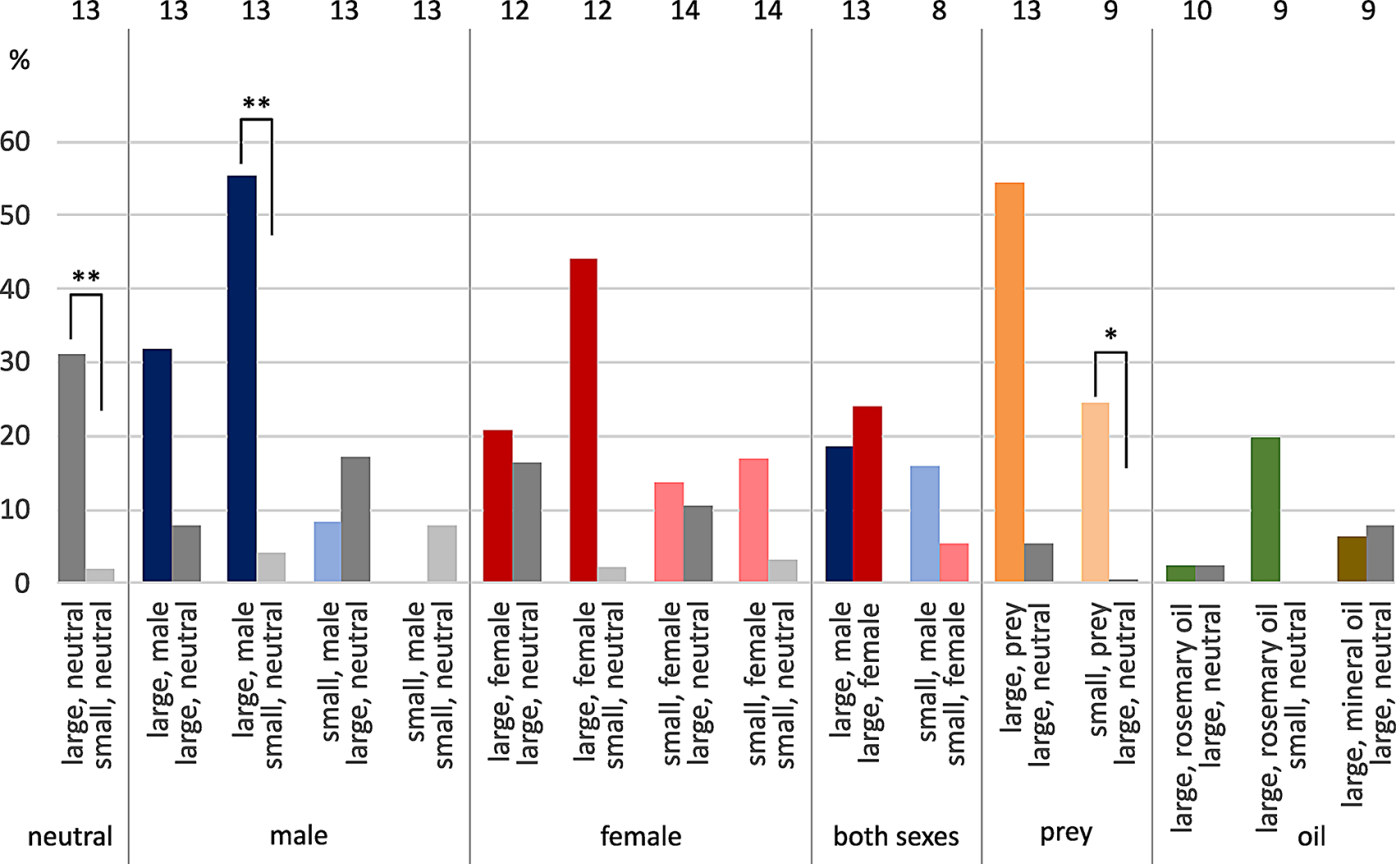
Median total times in contact with shelters in female *M. eupeus*. Ordinate, percent of total observation time. Tests are sorted by stimulus (neutral, male, female, both sexes, prey, rosemary oil, mineral oil) and shelter size (large-large, large-small, small-large, small-small). Statistically significant differences indicated by asterisks: * *P* < 0.05, ** *P* < 0.01. Error bars omitted for clarity (see Table S5). Sample sizes on top. Color codes: grey = neutral, blue = male, red = female, orange = prey, green = rosemary oil, brown = mineral oil; dark colors = large shelters, light colors = small shelters

Females of *M. eupeus* spent significantly longer median total times in contact with “large, neutral” versus “small, neutral” (paired t-test: t = 3.366, *n* = 13, *P* = 0.006, Fig. 6). Further, the combination of “large, male” versus “small, neutral” was statistically significant in favor of “large, male” (paired t-test: t = 3.434, *n* = 13, *P* = 0.005). Additionally, females spend significantly longer total median time in contact with a small but prey-scented shelter versus a large neutral one (paired t-test: t = -3.082, *n* = 9, *P* = 0.015).

### *M. eupeus*, median times “on top” and “underneath”

**Fig. 7:**
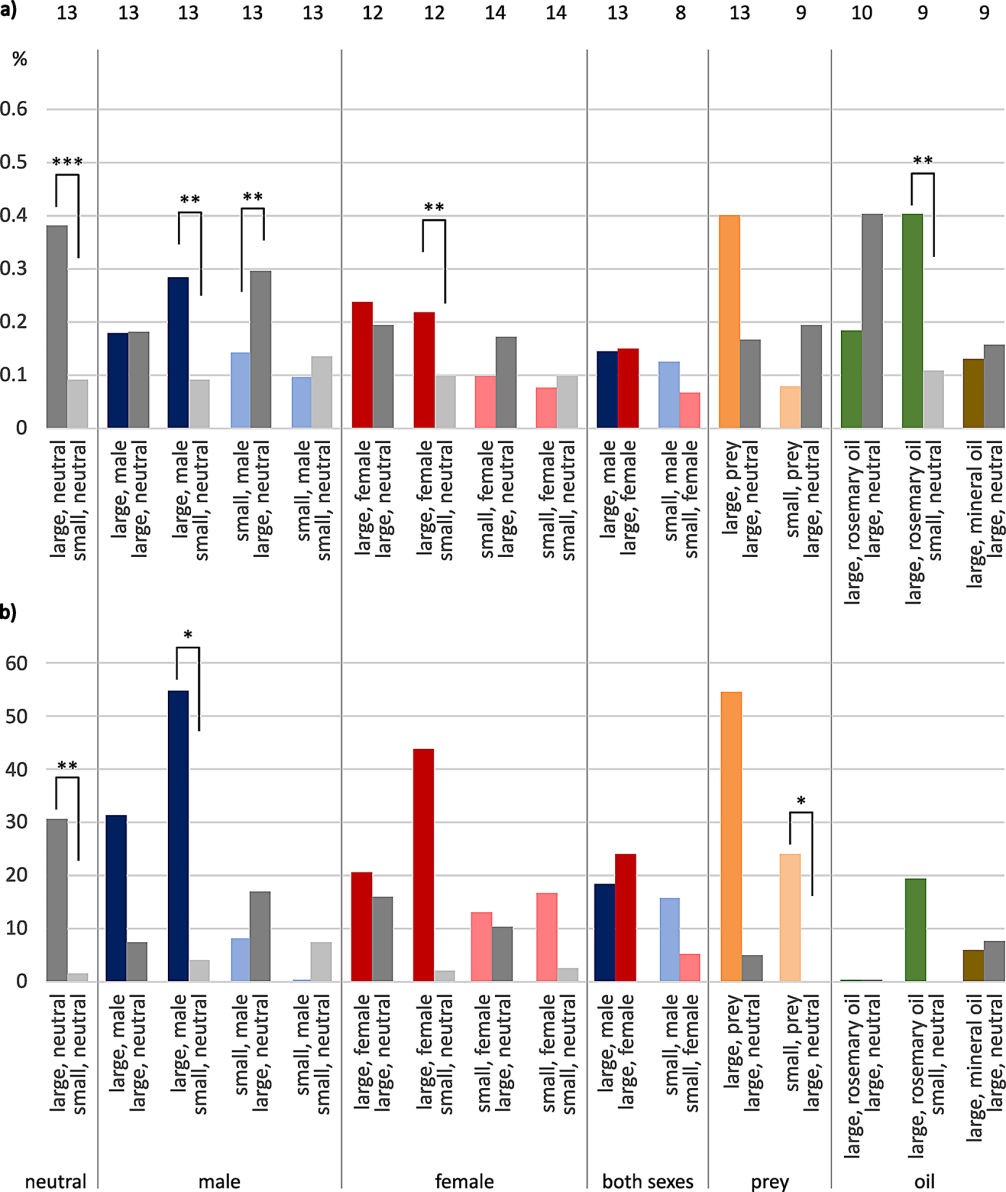
Median times in contact with shelter in female *M. eupeus*, plotted separately for (**a**) times on top of shelter and (**b**) times underneath shelter. Ordinates percent of total observation time. Tests are sorted by stimulus (neutral, male, female, both sexes, prey, rosemary oil, mineral oil) and shelter size (large-large, large-small, small-large, small-small). Statistically significant differences indicated by asterisks: * *P* < 0.05, ** *P* < 0.01, *** *P* ≤ 0.001. Error bars omitted for clarity (see Table S5). Sample sizes on top. Color codes: grey = neutral, blue = male, red = female, orange = prey, green = rosemary oil, brown = mineral oil; dark colors = large shelters, pale colors = small shelters

For the median contact times spend on top of hiding places, statistically significant differences were present in favor of “large, neutral” in comparison to “small, neutral” (Wilcoxon Signed Rank Test: W = -91.000, *n* = 13, *P* < 0.001; Fig. 7a, Table S6). Tested animals also spent longer median times on top of “large, male” than on “small, neutral” (paired t-test: t = 3.753, *n* = 13, *P* = 0.003). The same held true when the large hideout was female-scented (paired t-test: t = 3.619, *n* = 13, *P* = 0.004). Median times spent on top of “large, neutral” were also significantly longer compared to “small, male” (paired t-test: t = -3.340, *n* = 13, *P* = 0.006). The individuals spent even more median time on top of “large, rosemary oil” than on top of “small, neutral” (Wilcoxon Signed Rank Test: W = -43.000, *n* = 9, *P* = 0.008). As the times on top of shelters ranged in minutes and therefore represent less than 1% of the total time, there were no notable differences between the median total times and median times spend underneath a shelter (Fig. 7b).

### *M. eupeus*, impaired sensory organs

**Fig. 8:**
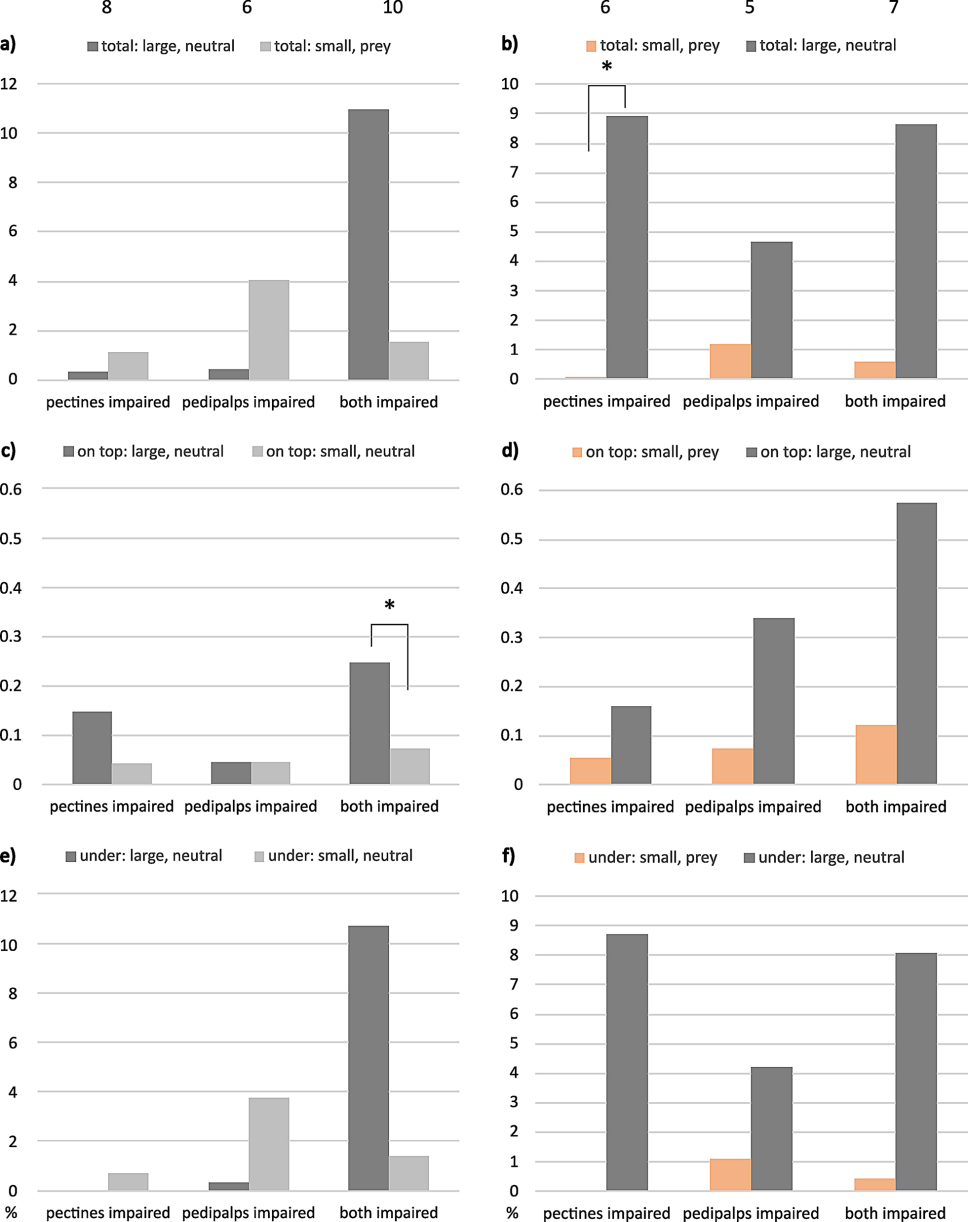
Median contact times of *M. eupeus* females with impaired sensory organs. Ordinates, percent of total observation time. (**a**, **b**) Total time in contact with shelter, (**c**, **d**) time on top of shelter, (**e**, **f**) time spent underneath shelter. (**a**, **c**, **e**) Left column, neutral test large versus small; (**b**, **d**, **f**) right column, “small, prey” tested versus “large, neutral”. Statistically significant differences indicated by asterisks: * *P* < 0.05. Error bars omitted for clarity (see Table S7). Sample sizes on top. Color codes: grey = neutral, orange = prey scent; dark colors = large shelters, light colors = small shelters

The maxima of median total contact times with impaired sensory organs in *M. eupeus* ranged below 12% of total observation time (Fig. 8). Only when both, pedipalps and pectines were severed, tested females showed a tendency toward longer median total contact times with “large, neutral” compared to “small, neutral”. This tendency became significant for longer times spend on top the large shelter compared to the small shelter (0.2%: 0.1%, *n* = 10, *P* = 0.014, Table S8). Animals spent more time at the large neutral hideout than at the prey-scented one with impaired pectines (8.9%: 0.1%, *n* = 6, *P* = 0.031) as well as when both organs had been impaired (8.6%: 0.6%, *n* = 7).

There was a general difference between the two scorpion species tested. Female *M. eupeus* investigated the shelters for longer median times by hiding underneath (see Figs. 4b and 7b), and thus also showed longer median total times (see Figs. 3 and 6). *E. italicus*, by contrast, spent longer median times on top of the shelters, while showing shorter median total contact times.

### Congruence total contact time and final hiding place

Finally, we compared for congruence the total contact times per shelter with the final hiding place per animal, if it had chosen a final hiding place. We expected the total contact time for the final hiding shelter to be longer than the total time in contact with the competing shelter. It turned out that, if a scorpion had chosen a final hiding place, it was the shelter the animal had had the longest contact with in 85.6% of the observations for intact *E. italicus* and in 87.7% for intact *M. eupeus*.

## Discussion

**Fig. 9:**
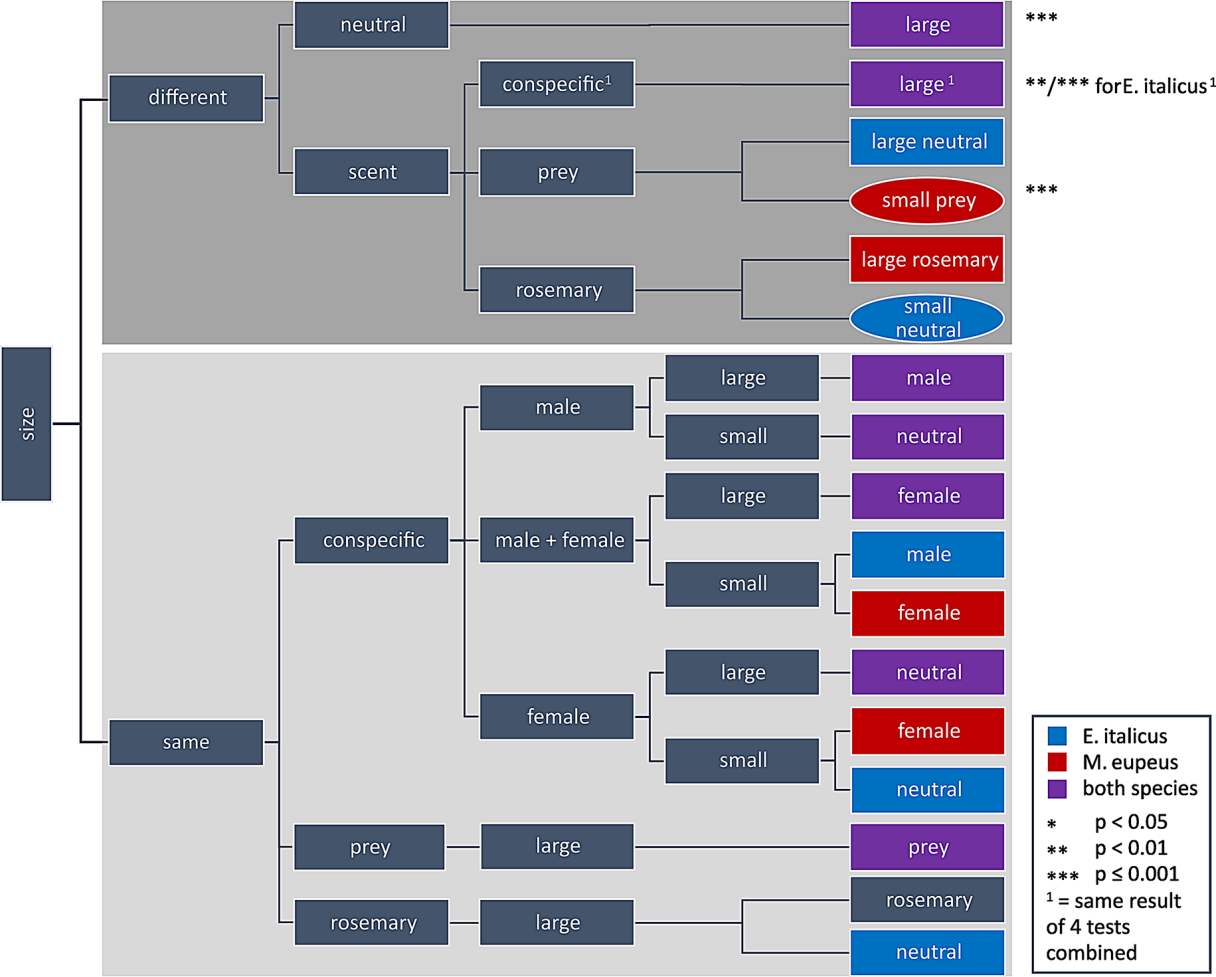
Decision tree of shelter choice in females of the two scorpion species. Animals are confronted with either two identical shelter sizes or the choice between large and small sizes. Size is also the first relevant information for tested individuals, as they preferred large over small shelters in neutral test situations, as well as when conspecific scent was added. For the latter case, the results of 4 different test situations are combined into one end node in the tree (^1^). For every combination of shelter size (same sizes, small-large, large-small), the decision path is further split by different types of additional scent: none = neutral, conspecific, prey and rosemary (oil). Only one shelter of a test pair was scented and tested against a neutral one of either same or different size. Except for the direct comparison of male vs. female scent where each of the shelters were scented with one of the conspecific scents. Final choice of the scorpions is marked by color coding: blue = *E. italicus*, red = *M. eupeus*, purple = both species. Most of the decisions mark tendencies, decisions that showed statistical significance are marked by asterisk: * *P* < 0.05, ** *P* < 0.01, *** *P* ≤ 0.001. Elliptical shapes highlight decisions where a small instead of a large shelter was preferred. In case of large rosemary scented shelters tested against large neutral shelters, tested females of *M. eupeus* did not show a clear preference and are thus omitted

We drew up a **decision tree** that summarizes the final shelter choice by the two scorpion species examined (Fig. 9). The first important aspect in shelter choice appears to be size, as both tested species significantly preferred large neutral over small neutral shelters (see below). Furthermore, tested females of *E. italicus* showed a clear tendency toward choosing larger shelters, independent of conspecific or prey scents. Females of *M. eupeus* also choose larger over smaller shelters, although not as independent of conspecific scent as *E. italicus*. Differently to *E. italicus*, *M. eupeus* significantly weighed prey scent over shelter size, which shows that chemical cues may override the preference for large shelter size. This holds true for the apparent avoidance of rosemary oil scent by female *E. italicus*, who preferred smaller shelters if only these are unperfumed. *M. eupeus*, by contrast, stuck to the larger shelters independent of added rosemary scent. In direct comparison of female vs. male scents, tested females of both species were likely to prefer the conspecific female scent in their final hiding place if both shelters were large ones. Intriguingly, in a large-large comparison both species tended to hide under the shelter perfumed with male scent instead of a neutral one and they chose neutral over female scent, decisions being more pronounced in *M. eupeus*. This behavior may be influenced by social context which will be discussed below.

In summary, scorpions tend to choose **larger shelters**. This may be due to a more stable and favorable microclimate regarding moisture, temperature and other parameters (Kaltsas et al. 2009; Becker and Brown 2016). An increasing number of animals that do not accept shelters in small vs. small comparisons supports this interpretation, as does the tendency of female (sand) scorpions to inhabit larger burrows than males (Williams 1966; Polis et al. 1986). The female preference for more space may be associated with being the larger sex (Braunwalder 2005; Kovařík et al. 2022) and with the maternal behavior of carrying offspring on the back until the larvae have molted for the first time (Shorthouse and Marples 1982; own observations).

In both species, the shelter that obtained the longer total **contact time** was also the final hiding place in most of the cases. In general, scorpions spent only short time periods on top of shelters (Figs. 4, 5, 7 and 8). We thus conclude that times spend underneath shelters are crucial for shelter rating. “On top” may provide a first impression of shelter suitability in terms of substrate structure, size and potential inhabitant odor cues, whereas “underneath” should provide exact information about space and animals currently hiding there.

### Conspecific scent & seasonality

It has been suggested for *Hadrurus arizonensis*, *A. gibbosus*, and *Paruroctonus boreus* that males actively search for females to mate, following female scent trails (compare: Melville et al. 2003; Kaltsas et al. 2009; Miller and Formanowicz 2011). It may thus be assumed that tested females select shelters with favorable properties, as noted above, regardless of (male) scent. Here, they may rest during daytime and wait for potential mating partners. Benton (1993) observed mating behavior in *E. flavicaudis*, and the majority of mating events took place in the female’s shelter, some males even cohabited in the female’s shelter prior to mating (Benton 1992b). This cohabitation is called “mate guarding” and is typically initiated by the male entering a female’s shelter, mostly while the residential female still carries its current clutch of larvae. Mate guarding lasted an average 10 days, until the females became receptive (Benton 1992b). Indeed, most authors assume that mating in *E. flavicaudis* follows the breeding season, as soon as the offspring have left their mothers (Benton 1992a, b, 1993; Braunwalder 2005). *E. italicus* may well show similar mating behavior, rendering female preference for larger shelters ever more appropriate.

Another aspect that may be relevant in this context is indicated by the study of Miller and Formanowicz (2011). They observed that male *Paruroctonus boreus* differentiate between females from their own and from a different population. The males showed actually little or no interest in the latter. It would thus be an interesting point for further study to address shelter choice in relation to population dynamics.

Seasonality also plays a role in shelter selection independent of reproductive behavior. In winter, *A. gibbosus* is apparently more social since found in groups more often (Kaltsas et al. 2009). Forming larger groups may facilitate dealing with harsh climatic conditions, or it may result more from aggregation in shelters with more favorable conditions, such as the microclimate under large stones. As scorpions reduce their activity level during winter, reduced aggression levels may allow denser shelter occupation. Likewise, *M. eupeus* shows an activity peak during higher temperatures (Fet 1980; Kassiri et al. 2015; Nejati et al. 2017). We examined *M. eupeus* under artificial mild summer conditions with stable temperatures, which may have biased choices toward roughly equal distribution between large and small shelters. Part of the scorpions in the experimental population may have been in the reproductive phase, preferring a neutral shelter, another part in hibernation mode, favoring shelters with conspecific scent.

Cannibalism or lethal territorial conflicts may occur in both examined scorpion species (Colombo 2009; Dehghani et al. 2016), though is rare when there are more shelters than animals and when food is abundant. However, there appears to be an influence of some kind of competition between *M. eupeus* females, as indicated by their higher interest in conspecific female-scented shelters during the night, while favoring a neutral final shelter of the same size for the daytime, avoiding female scent for the resting phase.

### Rosemary & prey scent

In shelter decisions, prey scent appears to be attractive to *M. eupeus* and to some extent to *E. italicus*, too, whereas rosemary oil acts as a repellent to *E. italicus* but does not appear to influence *M. eupeus*.

*E. italicus* lives in moderate climates, where prey abundance is typically rather high. The scent of crickets or other insect prey would thus appear to be of interest during the scorpions’ active phase only, and if they are hungry. During daytime, the presence of insects might disturb scorpions as they rest in their shelters. *E. italicus* may thus dislike a hideout with prey and prefer an empty one, as demonstrated previously for *Leiurus quinquestriatus* (Abushama 1964). Moreover, *E. italicus* may be alerted to prey more by vibratory rather than chemical cues, as suggested for other scorpion species previously (Brownell 1984; Mineo and Del Claro 2006).

*M. eupeus* lives in semi-arid habitats where prey is comparatively scarce. Compared to *E. italicus*, this species may thus have to search for prey more actively, in keeping with the generally more active life style of Buthidae (Warburg 2013). This agrees with the longer activity phase of *M. eupeus* during the night: *M. eupeus* is hunting for food or searching for mates between 9 pm and 1 am (Fet 1980), whereas *Euscorpius* shows activity peaks only during dusk and dawn (Wuttke 1966; Benton 1992a). Further, males and females may follow different hunting strategies, as reported for *A. gibbosus* (Kaltsas et al. 2008). Males are considered to hunt in their burrow’s surrounding in this species, whereas females are more likely to wait for prey in or near their burrow entrance. In conclusion, the response to prey scent appears to be dependent on species, sex, and habitat. This puts our present results in *M. eupeus* in context, suggesting that females are especially interested in prey-scented shelters, where hunting success with little energy expenditure may be relatively high.

Kelley and colleagues (Kelley et al. 2019) have reported aversive reactions of the New World scorpion *Centruroides vittatus* to rosemary oil, similar to our observations in *E. italicus*. Rosemary oil is a secondary plant component acting as insect repellent and thus protecting against insect herbivores (Isman et al. 2008). Either, this repellent effect extends beyond insects to other arthropod groups, including selected scorpion species. Or, the effect of rosemary oil is more indirect, alerting scorpions to areas with reduced insect abundance and thus fewer prey (Colombo 2006). These two options are not exclusive, of course. *M. eupeus*, living in semi-arid climates, may not need to respond to insect-aversive secondary plant compounds, at least not those of rosemary. Insect abundance is low to begin with in *M. eupeus*’ habitats, and so is the abundance of leafy herbs that need to rely on secondary compound repellents. This may be the reason for choosing shelters by size rather than (absence of) scent.

In summary, it appears that scorpion species’ responses to rosemary oil are part of the species’ adaptations to different habitats. The unique chemosensory appendages of scorpions, the pectines, are suggested to reflect such adaptations by their different morphologies, including numbers of pegs and chemosensory peg sensilla (Drozd et al. 2020).

### Impaired sensory organs

The results discussed above were the basis for our effort to identify the primary chemosensory organs involved in shelter evaluation. Severing both pectines and/or covering the pedipalps with acrylic paint resulted in the loss of differentiation between large and small shelters, and the number of animals that did not hide increased remarkably (compare Figs. 1 and 2). Apparently, impairment of pectines and/or pedipalps abolished sensory input necessary for shelter choice or for finding and identifying a shelter in the first place.

Most of the animals not hidden were those with severed pectines, suggesting that the pectines are the most important structures for shelter identification and selection. This is not surprising when considering the role of pectines in a broad spectrum of behaviors. Pectines receive both mechano- and chemosensory input (Cloudsley-Thompson 1955; Foelix and Müller-Vorholt 1983; Brownell 1989; Gaffin and Brownell 1997; Wolf 2008, 2017; Knowlton and Gaffin 2011). Besides their suggested role in detection of vibrations (Brownell 1984; Mineo and Del Claro 2006), obstacle avoidance (Schneider et al. 2003; Kladt et al. 2007; Drozd et al. 2022) and navigation (Gaffin and Brayfield 2017), the pectines also receive chemical signals from the substrate that are used for trailing mates or prey (Krapf 1986; Gaffin and Brownell 1992, 2001; Melville et al. 2003; Steinmetz et al. 2004; Taylor et al. 2012). The pectines should thus be equally suited to explore the substrate and associated scents of potential shelters. This interpretation is supported by the results in *E. italicus* with only the pedipalps impaired, where individuals chose a smaller but neutral shelter over a large, rosemary-scented hide-out (Fig. 2b). Detection of chemical compounds appears to be mediated also via hairs on the pedipalps (Steinmetz et al. 2004; Fet et al. 2006), probably primarily of airborne scents (Nisani et al. 2018; Maurer *Master Thesis* Ulm University).

In summary, scorpion pectines and pedipalps are mechano- and chemosensory organs of major importance. It is thus little surprise that shelter choice, and indeed acceptance of a shelter, is compromised by impairment of either pectines or pedipalps, or both. However, the apparent ability of adequate shelter choice in animals with both, pectines and pedipalps impaired, is intriguing (Fig. 2). Two sensory systems may contribute to this feat. These are mechano- and chemosensory sensilla on the tarsi (Foelix and Schabronath 1983) and mechanosensory slit organs in all the legs (Pringle 1955; Barth and Wadepuhl 1975; Barth and Stagl 1976). The latter have been shown to be employed for path integration in spiders, where these organs are embraced by the term lyriform organs (Moller and Görner 1994; Nørgaard et al. 2003; Barth 2021). A similar function of these sensilla has been hypothesized also for scorpions (Prévost and Stemme 2020). These strain receptors might thus be involved in distance measuring and consequently in the evaluation of shelter size. For *Leiurus quinquestriatus*, as well as the desert sand scorpion *Smeringurus mesaensis*, it has been shown that they rely on their tarsal sensilla to detect moist substrates (Abushama 1964; Gaffin et al. 1992). As larger shelters seem more stable concerning temperature and humidity, the tarsal sensilla might explain part of the restored ability to consider the larger shelter as favorable.

With pectines and pedipalps serving as primary organs for identification and choice of shelters, input from tarsal sensors and path integration networks would become relevant only when those primary organs are impaired.

### Summary and perspectives

The data presented here contribute insights for the prevention of harmful human-scorpion encounters, as they form the base for a better understanding of scorpion shelter selection, including urban regions. Females of both *E. italicus* and *M. eupeus* favor larger over smaller shelters, while they do not show clear preferences for conspecific scents. However, *E. italicus* is repelled by rosemary oil scent, and *M. eupeus* attracted by prey scent. Thus, we were able to influence the general choice of the scorpions for a larger shelter toward a smaller shelter by adding attractive or aversive scents, indicating that scent is important for shelter choice, both as aversive or attractive cue. These effects are so pronounced that scorpions choose the smaller shelters if those carry the preferred scent. Responses to scents are thus species-specific and may be related to the different habitats, namely, moderate versus semi-arid climates. The primary chemosensory organs, namely pectines and pedipalps, perceive scents associated with hideouts, and are involved in shelter evaluation. However, results of experiments with both organs being impaired – namely the partial recovery of results of intact animals - leave us with more questions than answers. Future work should investigate the role of other chemosensory sensilla, for example tarsal mechano- and chemosensors that may compensate impairment of the primary chemosensory organs. Including a broader species sample in future studies, especially from desert and rain forest habitats, may further be commendable. Influence of population dynamics on female shelter selection would be of interest, too. Our pioneering work will serve as a solid basis for these and related continuing research strands.

## Electronic supplementary material

Below is the link to the electronic supplementary material.

Supplementary Material 1

## Data Availability

The data that support the findings of this study are available from corresponding authors upon reasonable request.
