# Supplementary material for: Shelter selection in females of two scorpion species depends on shelter size and scent
Source: J Comp Physiol A Neuroethol Sens Neural Behav Physiol. 2024 Nov 2;211(2):163–83. doi: 10.1007/s00359-024-01721-6 (PMC12003491; doi:10.1007/s00359-024-01721-6)
Supplement: Supplementary file 1 — Supplementary Material 1 [file 359_2024_1721_MOESM1_ESM.pdf]

Shelter selection in females of two scorpion species depends on shelter size and scent

Journal of Comparative Physiology A

Janina Hladik*, Yorick Bailer, Harald Wolf*, Torben Stemme*

Institute of Neurobiology, Ulm University, Albert-Einstein-Allee 11, 89081 Ulm, Germany

**Corresponding authors.*

E-Mail: janina.hladik@uni-ulm.de, harald.wolf@uni-ulm.de, torben_stemme@gmx.net

ORCIDs: HW: 0000-0003-2715-3376, TS: 0000-0003-2751-2690

Table S1 Median contact times, standard deviations S, and standard errors (hh:mm:ss) for *E. italicus* in the different test situations, intact animals

shelter combination	median time underneath	standard deviation S	standard error median	median time on top	standard deviation S	standard error median	median total contact time	standard deviation S	standard error median
large, neutral	05:59:38	02:59:00	00:54:25	00:01:50	00:01:21	00:00:26	06:00:47	02:58:44	00:57:50
small, neutral	00:03:24	01:48:40	00:35:10	00:02:12	00:01:07	00:00:22	00:05:43	01:48:40	00:35:10
large, male	00:42:04	04:18:32	01:18:35	00:04:28	05:54:54	01:47:53	02:09:54	06:42:33	02:02:22
large, neutral	00:41:01	04:00:28	01:13:06	00:03:29	00:04:09	00:01:16	00:43:39	03:59:21	01:12:45
large, male	03:26:27	03:47:31	01:09:10	00:03:14	10:59:10	03:20:22	04:54:03	11:16:13	03:25:33
small, neutral	00:00:00	01:07:13	00:20:26	00:01:47	00:04:04	00:01:14	00:10:44	01:05:49	00:20:01
small, male	00:00:00	02:04:42	00:36:50	00:01:53	00:01:37	00:00:29	00:02:58	02:05:00	00:36:56
large, neutral	06:16:05	04:17:21	01:16:01	00:03:16	00:02:45	00:00:49	06:19:17	04:16:22	01:15:44
small, male	00:08:13	02:22:26	00:40:57	00:02:28	00:02:41	00:00:46	00:08:56	02:21:48	00:40:46
small, neutral	00:39:19	02:45:23	00:47:33	00:02:27	00:02:18	00:00:40	00:41:40	02:44:40	00:47:21
large, female	01:38:55	02:57:57	00:54:06	00:04:35	00:02:02	00:00:38	01:43:40	02:57:36	00:55:39
large, neutral	01:30:19	03:59:24	01:15:01	00:04:14	00:03:13	00:01:01	01:34:47	03:58:25	01:14:42
large, female	04:34:15	03:52:52	01:15:21	00:03:36	00:02:34	00:00:50	04:37:21	03:52:24	01:15:12
small, neutral	00:00:00	00:42:47	00:13:51	00:02:01	00:01:37	00:00:31	00:03:30	00:42:27	00:13:44
small, female	00:00:00	08:04:50	02:19:24	00:02:06	00:03:27	00:01:00	00:02:17	08:04:17	02:19:15
large, neutral	05:52:22	03:23:57	00:58:39	00:02:12	00:04:11	00:01:12	05:54:56	03:23:06	00:58:24
small, female	00:04:37	02:18:20	00:38:46	00:02:29	00:03:42	00:01:02	00:10:11	02:17:59	00:38:40
small, neutral	00:01:37	02:39:07	00:44:36	00:03:38	00:03:01	00:00:51	00:10:31	02:38:36	00:44:27
large, male	00:13:51	03:31:27	01:02:28	00:03:19	00:05:21	00:01:35	00:29:21	03:29:36	01:01:55
large, female	00:50:44	04:26:29	01:18:43	00:02:35	00:03:28	00:01:01	00:58:43	04:25:06	01:18:19
small, male	00:00:00	01:10:29	00:19:45	00:02:18	00:03:16	00:00:55	00:02:35	01:10:46	00:19:50
small, female	00:00:11	01:46:52	00:29:57	00:02:03	00:05:58	00:01:40	00:08:38	01:47:44	00:30:12
large, prey	02:21:26	03:34:36	01:01:42	00:03:53	00:03:36	00:01:02	02:25:19	03:33:31	01:01:23
large, neutral	01:37:42	03:39:01	01:02:58	00:02:51	00:06:40	00:01:55	01:38:59	03:37:24	01:02:30
small, prey	00:00:00	01:18:15	00:22:30	00:01:17	00:01:48	00:00:31	00:02:10	01:18:10	00:22:29
large, neutral	00:08:53	02:33:47	00:44:13	00:02:47	00:05:40	00:01:38	00:25:10	02:31:41	00:43:37
large, rosemary oil	00:00:00	02:56:58	00:52:17	00:01:08	00:02:44	00:00:48	00:02:24	02:56:54	00:52:15
large, neutral	00:02:03	03:36:21	01:03:55	00:02:32	00:56:15	00:16:37	00:26:13	03:35:50	01:03:46
large, rosemary oil	00:00:00	00:21:32	00:06:45	00:01:15	00:06:22	00:02:00	00:01:30	00:23:04	00:07:14
small, neutral	00:01:16	03:20:02	01:02:41	00:02:40	00:03:38	00:01:08	00:08:39	03:18:37	01:02:14
large, mineral oil	00:00:00	02:27:10	00:44:44	00:02:40	00:02:20	00:00:42	00:04:05	02:27:03	00:44:42
large, neutral	00:36:39	03:57:39	01:12:15	00:02:51	00:03:57	00:01:12	00:38:39	03:57:57	01:12:20

Table S2 Statistical results for median contact times in *E. italicus*, intact animals. For each test situation, results of paired t-test (= t, when normality test passed) or Wilcoxon signed-rank test (= W, when normality test failed), and corresponding P-values are listed. Significant P-values are highlighted in bold. If the desired power of a t-test with alpha = 0.050 was below 0.800, power value is noted. Additionally, results of Fisher exact test for the final shelter choices are shown

	median time underneath		median time on top		median total contact time			Fisher Exact	
shelter combination	t / W	P	t / W	P	t / W	P	n	P	n
large, neutral			alpha = 0.050: 0.262				15	**<0.001**	15
small, neutral	t = -3.890	**P = 0.002**	t = -1.768	P = 0.099	t = -3.892	**P = 0.002**			
large, male	alpha = 0.050: 0.050		W= 65.000	P = 0.132	alpha = 0.050: 0.050		17	0.466	15
large, neutral	t = -0.124	P = 0.903			t = -0.741	P = 0.469			
large, male			W= 69.000	P = 0.109	W= 127.000	**P = 0.001**	17	**<0.001**	16
small, neutral	t = -3.916	**P = 0.001**							
small, male			W= -133.000	**P = 0.002**			18	**0.005**	17
large, neutral	t = 3.624	**P = 0.002**			t = 3.643	**P = 0.002**			
small, male	W= -46.000	P = 0.208	alpha = 0.050: 0.050		W= -60.000	P = 0.241	19	0.74	18
small, neutral			t = -0.725	P = 0.478					
large, female	alpha = 0.050: 0.050		alpha = 0.050: 0.050		alpha = 0.050: 0.050		16	1,000	15
large, neutral	t = 0.534	P = 0.601	t = 0.435	P = 0.670	t = 0.538	P = 0.599			
large, female							15	**0.001**	13
small, neutral	t = -3.445	**P = 0.004**	t = -4.475	**P = <0.001**	t = -3.477	**P = 0.004**			
small, female	W= -95.000	**P = 0.023**	W= -138.000	**P = 0.004**	W= -122.000	**P = 0.012**	19	**<0.001**	18
large, neutral									
small, female	alpha = 0.050: 0.050		alpha = 0.050: 0.050		alpha = 0.050: 0.050		20	0.494	17
small, neutral	t = -0.153	P = 0.880	t = -0.0151	P = 0.988	t = -0.154	P = 0.879			
large, male	alpha = 0.050: 0.050		W= 73.000	P = 0.119			18	0.076	16
large, female	t = 0.477	P = 0.639			t = 0.461	P = 0.651			
small, male	W= -46.000	**P = 0.042**	W= -24.000	P = 0.674	W= -120.000	**P = 0.024**	20	0.257	14
small, female									
large, prey	alpha = 0.050: 0.050		W= -24.000	P = 0.651	alpha = 0.050: 0.050		19	0.494	17
large, neutral	t = 0.116	P = 0.909			t = 0.131	P = 0.897			
small, prey	alpha = 0.050: 0.276		W= -152.000	**P = 0.001**	alpha = 0.050: 0.308		19	0.076	16
large, neutral	t = 1.790	P = 0.090			t = 1.871	P = 0.078			
large, rosemary oil	W= -12.000	P = 0.638	W= -129.000	**P = 0.003**	W= -63.000	P = 0.181	18	0.115	13
large, neutral									
large, rosemary oil	W= -36.000	P = 0.176	W= -12.000	P = 0.782	W= -60.000	P = 0.130	16	0.257	14
small, neutral									
large, mineral oil	alpha = 0.050: 0.175		alpha = 0.050: 0.278		alpha = 0.050: 0.180		17	0.706	14
large, neutral	t = 1.506	P = 0.152	t = 1.801	P = 0.091	t = 1.523	P = 0.147			

Table S3 Median contact times, standard deviations S, and standard errors (hh:mm:ss) for *E. italicus* in the different test situations, impaired animals

impaired organ	shelter combination	median time underneath	standard deviation S	standard error median	median time on top	standard deviation S	standard error median	median total contact time	standard deviation S	standard error median
pectines	small, neutral	00:01:59	00:03:48	00:01:30	00:01:59	00:03:48	00:01:30	00:00:00	00:00:00	00:00:00
	large, rosemary oil	00:02:33	01:31:57	00:36:26	00:01:59	00:01:55	00:00:46	00:00:00	01:30:34	00:35:54
pedipalps	small, neutral	00:01:30	03:46:34	01:47:20	00:00:47	00:01:12	00:00:34	00:00:00	03:47:07	01:47:35
	large, rosemary oil	00:01:14	00:02:25	00:01:09	00:01:14	00:02:25	00:01:09	00:00:00	00:00:00	00:00:00
both	small, neutral	00:01:36	01:58:01	00:36:59	00:01:15	00:07:26	00:02:20	00:00:00	01:58:03	00:36:59
	large, rosemary oil	00:01:52	00:25:05	00:07:52	00:01:52	00:01:58	00:00:37	00:00:00	00:24:21	00:07:38
pectines	small, neutral	00:04:02	01:52:44	00:47:06	00:02:16	00:01:36	00:00:40	00:00:00	01:52:01	00:46:48
	large, neutral	00:07:10	03:00:57	01:15:36	00:04:51	00:02:42	00:01:08	00:00:00	03:01:21	01:15:46
pedipalps	small, neutral	00:21:21	04:01:52	01:54:34	00:01:28	00:01:07	00:00:32	00:18:40	04:01:50	01:54:33
	large, neutral	00:02:54	01:15:45	00:35:53	00:01:15	00:01:03	00:00:30	00:00:00	01:16:09	00:36:04
both	small, neutral	00:03:38	02:40:32	00:50:18	00:01:24	00:02:13	00:00:42	00:00:00	02:40:56	00:50:26
	large, neutral	00:07:46	02:17:16	00:43:00	00:02:47	00:03:26	00:01:05	00:00:00	02:18:06	00:43:16

Table S4 Statistical results for median contact times in *E. italicus*, impaired animals. For each test situation, results of paired t-test (= t, when normality test passed) or Wilcoxon signed-rank test (= W, when normality test failed), and corresponding P-values are listed. Significant P-values are highlighted in bold. If the desired power of a t-test with alpha = 0.050 was below 0.800, power value is noted. Additionally, results of Fisher exact test for the final shelter choices are shown

impaired organ	shelter combination	median time underneath		median time on top		nedian total contact time			Fisher Exact	
		t / W	P	t / W	P	t / W	P	n	P	n
pectines	small, neutral	W= 1.000	P = 1.000	alpha = 0.050: 0.269		W= 5.000	P = 0.820	9	P = 0.206	5
	large, neutral			t = -1.851	P = 0.101					
pedipalps	small, neutral	alpha = 0.050: 0.050		alpha = 0.050: 0.050		alpha = 0.050: 0.050		7	P = 1.000	7
	large, neutral	t = 0.927	P = 0.390	t = -0.908	P = 0.399	t = 0.925	P = 0.391			
both	small, neutral	W= 5.000	P = 0.820	ıa = 0.050: 0.277		W= 28.000	P = 0.495	16	**P = 0.039**	12
	large, neutral			t = -1.805	P = 0.091					
pectines	small, neutral	W= 3.000	P = 0.500	W= -5.000	P = 0.846	W= 11.000	P = 0.625	10	P = 1.000	4
	large, rosemary oil									
pedipalps	small, neutral	W= -3.000	P = 0.500	alpha = 0.050: 0.154		W= -12.000	P = 0.375	7	P = 0.080	6
	large, rosemary oil			t = -1.510	P = 0.182					
both	small, neutral	W= -14.000	P = 0.297	W= 20.000	P = 0.632	W= -26.000	P = 0.528	16	P = 1.000	11
	large, rosemary oil									

Table S5 Median contact times, standard deviations S, and standard errors (hh:mm:ss) for *M. eupeus* in the different test situations, intact animals

shelter combination	median time underneath	standard deviation S	standard error median	median time on top	standard deviation S	standard error median	median total contact time	standard deviation S	standard error median
large, neutral	04:02:12	03:44:54	01:18:11	00:02:59	00:14:07	00:04:54	03:58:44	03:47:24	01:19:03
small, neutral	00:14:51	01:29:32	00:31:07	00:00:43	00:00:48	00:00:17	00:12:45	01:29:39	00:31:10
large, male	04:08:55	02:59:34	09:22:58	00:01:24	00:12:57	00:04:30	04:05:44	03:01:00	09:23:28
large, neutral	01:01:41	04:11:22	01:27:23	00:01:25	00:01:15	00:00:26	00:58:01	04:11:47	01:27:31
large, male	07:13:22	03:28:20	01:12:25	00:02:13	00:02:32	00:00:53	07:07:33	03:28:46	01:12:34
small, neutral	00:32:13	01:41:24	00:35:15	00:00:43	00:00:25	00:00:09	00:31:12	01:41:24	00:35:15
small, male	01:04:33	03:47:18	01:19:01	00:01:07	00:00:46	00:00:16	01:04:22	03:47:20	01:19:01
large, neutral	02:13:30	02:54:26	01:00:38	00:02:19	00:02:09	00:00:45	02:11:27	02:53:56	01:00:28
small, male	00:02:20	03:30:39	01:13:14	00:00:45	00:00:53	00:00:18	00:00:25	03:30:53	01:13:18
small, neutral	01:00:14	02:42:31	00:56:30	00:01:03	00:00:32	00:00:11	00:58:35	02:42:22	00:56:26
large, female	02:43:18	04:27:40	01:36:50	00:01:51	00:01:38	00:00:35	02:40:54	04:27:39	01:36:50
large, neutral	02:07:45	04:06:19	01:29:07	00:01:31	00:02:01	00:00:44	02:05:20	04:05:42	01:28:54
large, female	05:43:52	04:07:05	01:29:24	00:01:42	00:01:53	00:00:41	05:41:47	04:07:50	01:29:40
small, neutral	00:17:32	03:35:12	01:17:52	00:00:46	00:00:50	00:00:18	00:15:58	03:35:10	01:17:51
small, female	01:46:05	04:17:52	01:26:23	00:00:46	00:02:14	00:00:45	01:41:02	04:18:15	01:26:30
large, neutral	01:21:41	04:08:55	01:23:23	00:01:20	00:01:27	00:00:29	01:20:45	04:08:57	01:23:23
small, female	02:11:01	02:35:58	00:52:14	00:00:36	00:00:58	00:00:19	02:09:56	02:35:59	00:52:15
small, neutral	00:23:41	01:59:48	00:40:08	00:00:46	00:02:27	00:00:49	00:19:47	02:00:19	00:40:18
large, male	02:24:20	04:13:46	01:28:13	00:01:08	00:00:55	00:00:19	02:23:31	04:13:17	01:28:02
large, female	03:08:00	04:11:55	01:27:34	00:01:10	00:01:20	00:00:28	03:06:50	04:12:42	01:27:50
small, male	02:03:51	03:23:07	01:30:00	00:00:59	00:00:48	00:00:21	02:03:25	03:23:00	01:29:57
small, female	00:41:41	01:09:09	00:30:38	00:00:31	00:00:48	00:00:21	00:40:21	01:08:52	00:30:31
large, prey	07:05:35	04:27:42	01:33:03	00:03:08	00:04:34	00:01:35	07:05:30	04:26:54	01:32:47
large, neutral	00:40:55	03:10:02	01:06:03	00:01:18	00:01:11	00:00:25	00:39:01	03:10:21	01:06:10
small, prey	03:10:25	03:55:57	01:38:35	00:00:37	00:01:05	00:00:27	03:07:54	03:55:47	01:38:30
large, neutral	00:02:29	00:55:08	00:23:02	00:01:30	00:01:32	00:00:38	00:00:00	00:53:49	00:22:29
large, rosemary oil	00:19:38	02:41:18	01:03:56	00:01:26	00:11:06	00:04:24	00:00:33	02:44:10	01:05:04
large, neutral	00:18:00	01:34:01	00:37:16	00:03:08	00:27:26	00:10:52	00:02:22	01:36:02	00:38:03
large, rosemary oil	02:34:52	02:42:38	01:07:57	00:03:09	01:19:13	00:33:06	02:30:45	02:51:08	01:11:30
small, neutral	00:01:52	02:21:41	00:59:12	00:00:50	00:00:32	00:00:13	00:00:00	02:21:33	00:59:08
large, mineral oil	00:49:19	03:07:34	01:18:22	00:01:01	00:01:20	00:00:33	00:46:26	03:06:45	01:18:01
large, neutral	01:01:19	03:56:10	01:38:40	00:01:13	00:05:21	00:02:14	00:59:52	03:55:17	01:38:18

Table S6 Statistical results for median contact times in *M. eupeus*, intact animals. For each test situation, results of paired t-test (= t, when normality test passed) or Wilcoxon signed-rank test (= W, when normality test failed), and corresponding P-values are listed. Significant P-values are highlighted in bold. If the desired power of a t-test with alpha = 0.050 was below 0.800, power value is noted. Additionally, results of Fisher exact test for the final shelter choices are shown

	median time underneath		median time on top		median total contact time			Fisher Exact	
shelter combination	t / W	P	t / W	P	t / W	P	n	P	n
large, neutral			W= -91.000	**P = <0.001**			13	**<0.001**	12
small, neutral	t = 3.259	**P = 0.007**			t = 3.366	**P = 0.006**			
large, male	W= -35.000	P = 0.244	W= -50.000	P = 0.080	W= -35.000	P = 0.244	13	0.434	13
large, neutral									
large, male							13	**P = 0.003**	12
small, neutral	t = 3.394	**P = 0.005**	t = 3.753	**P = 0.003**	t = 3.434	**P = 0.005**			
small, male	alpha = 0.050: 0.050				alpha = 0.050: 0.050		13	P = 0.684	12
large, neutral	t = 0.109	P = 0.915	t = -3.340	**P = 0.006**	t = 0.0896	P = 0.930			
small, male	alpha = 0.050: 0.050		alpha = 0.050: 0.052				13	P = 0.395	11
small, neutral	t = -0.259	P = 0.800	t = 1.019	P = 0.328	t = -0.256	P = 0.802			
large, female	alpha = 0.050: 0.050		alpha = 0.050: 0.050		alpha = 0.050: 0.050		12	P = 0.220	12
large, neutral	t = 0.405	P = 0.693	t = 0.462	P = 0.653	t = 0.406	P = 0.693			
large, female	alpha = 0.050: 0.218				alpha = 0.050: 0.223		12	P = 1.000	11
small, neutral	t = 1.664	P = 0.124	t = 3.619	**P = 0.004**	t = 1.678	P = 0.122			
small, female	W= -37.000	P = 0.268	W= 37.000	P = 0.268			14	P = 0.684	12
large, neutral					t = 0.307	P = 0.764			
small, female	alpha = 0.050: 0.053		W= -9.000	P = 0.808	alpha = 0.050: 0.052		14	P = 0.086	11
small, neutral	t = 1.026	P = 0.324			t = 1.020	P = 0.326			
large, male	alpha = 0.050: 0.050		alpha = 0.050: 0.050		alpha = 0.050: 0.050		13	P = 0.115	13
large, female	t = 0.280	P = 0.785	t = 0.721	P = 0.484	t = 0.282	P = 0.783			
small, male	alpha = 0.050: 0.219		alpha = 0.050: 0.050		alpha = 0.050: 0.219		8	P = 1.000	7
small, female	t = 1.719	P = 0.129	t = 0.790	P = 0.455	t = 1.718	P = 0.129			
large, prey	alpha = 0.050: 0.307		W= -55.000	P = 0.057	alpha = 0.050: 0.315		13	P = 0.115	13
large, neutral	t = 1.902	P = 0.081			t = 1.923	P = 0.079			
small, prey			alpha = 0.050: 0.178				9	**P = <0.001**	8
large, neutral	t = -3.102	**P = 0.015**	t = 1.563	P = 0.157	t = -3.082	**P = 0.015**			
large, rosemary oil	alpha = 0.050: 0.050		W= -29.000	P = 0.160	alpha = 0.050: 0.050		10	P = 1.000	8
large, neutral	t = -0.829	P = 0.428			t = -0.763	P = 0.465			
large, rosemary oil	alpha = 0.050: 0.050		W= -43.000	**P = 0.008**	alpha = 0.050: 0.067		9	P = 0.619	8
small, neutral	t = 0.793	P = 0.451			t = 1.099	P = 0.304			
large, mineral oil	alpha = 0.050: 0.050		W= 7.000	P = 0.734	alpha = 0.050: 0.050		9	P = 1.000	9
large, neutral	t = 0.129	P = 0.900			t = 0.141	P = 0.892			

Table S7 Median contact times, standard deviations S, and standard errors (hh:mm:ss) for *M. eupeus* in the different test situations, impaired animals

impaired organ	shelter combination	median time underneath	standard deviation S	standard error median	median time on top	standard deviation S	standard error median	median total contact time	standard deviation S	standard error median
pectines	small, prey	00:00:27	00:21:22	00:10:56	00:00:26	00:00:38	00:00:20	00:00:00	00:21:30	00:11:00
	large, neutral	01:09:30	01:28:05	00:45:04	00:01:16	00:01:27	00:00:45	01:07:56	01:27:12	00:44:37
pedipalps	small, prey	00:09:13	01:14:17	00:41:38	00:00:34	00:01:22	00:00:46	00:08:39	01:13:30	00:41:12
	large, neutral	00:36:19	01:57:11	01:05:41	00:02:39	00:05:16	00:02:57	00:32:59	02:00:24	01:07:29
both	small, prey	00:04:28	04:58:53	02:21:35	00:00:57	00:00:39	00:00:19	00:03:22	04:59:06	02:21:41
	large, neutral	01:07:31	04:34:46	02:10:10	00:04:29	00:06:10	00:02:55	01:03:02	04:35:52	02:10:41
pectines	small, neutral	00:08:56	02:42:32	01:12:01	00:00:20	00:02:08	00:00:57	00:05:20	02:43:08	01:12:17
	large, neutral	00:02:42	03:42:16	01:38:29	00:01:09	00:01:56	00:00:51	00:00:00	03:42:20	01:38:31
pedipalps	small, neutral	00:31:49	02:07:15	01:05:07	00:00:21	00:01:13	00:00:37	00:29:22	02:07:37	01:05:18
	large, neutral	00:03:21	02:44:16	01:24:03	00:00:22	00:05:21	00:02:44	00:02:35	02:45:03	01:24:27
both	small, neutral	00:11:59	00:52:04	00:20:38	00:00:35	00:00:37	00:00:14	00:11:04	00:51:35	00:20:27
	large, neutral	01:25:18	02:32:28	01:00:26	00:01:56	00:03:56	00:01:33	01:23:23	02:31:27	01:00:01

Table S8 Statistical results for median contact times in *M. eupeus*, impaired animals. For each test situation, results of paired t-test (= t, when normality test passed) or Wilcoxon signed-rank test (= W, when normality test failed), and corresponding P-values are listed. Significant P-values are highlighted in bold. If the desired power of a t-test with alpha = 0.050 was below 0.800, power value is noted. Additionally, results of Fisher exact test for the final shelter choices are shown

impaired organ	shelter combination	median time underneath		median time on top		median total contact time		n	Fisher Exact	
		t / W	P	t / W	P	t / W	P	n	P	n
pectines	small, neutral	W= 4.000	P = 0.625	alpha = 0.050: 0.050		W= 6.000	P = 0.742	8	P = 1.000	7
	large, neutral			t = -0.659	P = 0.531					
pedipalps	small, neutral	alpha = 0.050: 0.050		W= 9.000	P = 0.438	alpha = 0.050: 0.050		6	P = 1.000	4
	large, neutral	t = 0.163	P = 0.877			t = 0.147	P = 0.889			
both	small, neutral	alpha = 0.050: 0.286		W= 47.000	**P = 0.014**	alpha = 0.050: 0.297		10	P = 0.206	5
	large, neutral	t = -1.881	P = 0.093			t = -1.911	P = 0.088			
pectines	small, prey	W= 6.000	P = 0.250	alpha = 0.050: 0.144		W= 21.000	**P = 0.031**	6	P = 1.000	3
	large, neutral			t = -1.495	P = 0.195					
pedipalps	small, prey	alpha = 0.050: 0.050		W= 9.000	P = 0.313	alpha = 0.050: 0.050		5	(P = 0.486)	4
	large, neutral	t = -0.557	P = 0.607			t = -0.601	P = 0.580			
both	small, prey	alpha = 0.050: 0.050		alpha = 0.050: 0.412		alpha = 0.050: 0.050		7	P = 1.000	5
	large, neutral	t = -0.126	P = 0.904	t = -2.313	P = 0.060	t = -0.154	P = 0.883			
